# Multicentre pilot study evaluation of lung ultrasound for the management of paediatric pneumonia in low-resource settings: a study protocol

**DOI:** 10.1136/bmjresp-2018-000340

**Published:** 2018-12-19

**Authors:** Jennifer L Lenahan, Giovanni Volpicelli, Alessandro Lamorte, Fyezah Jehan, Quique Bassat, Amy Sarah Ginsburg

**Affiliations:** 1 International Programs, Save the Children Federation Inc, Fairfield, Connecticut, USA; 2 Emergency Medicine, San Luigi Gonzaga University Hospital, Turin, Italy; 3 Emergency Medicine, Parini Hospital, Aosta, Italy; 4 Department of Paediatrics and Child Health, Aga Khan University, Karachi, Pakistan; 5 ISGlobal, Hospital Clínic - Universitat de Barcelona, Barcelona, Spain; 6 Centro de Investigação em Saúde de Manhiça (CISM), Maputo, Mozambique

**Keywords:** pneumonia, paediatric lung disaese, respiratory infection, imaging/CT MRI etc

## Abstract

**Introduction:**

Pneumonia is the leading infectious cause of death among children under 5 years of age worldwide. However, pneumonia is challenging to diagnose. Lung ultrasound (LUS) is a promising diagnostic technology. Further evidence is needed to better understand the role of LUS as a tool for the diagnosis of childhood pneumonia in low-resource settings.

**Methods and analysis:**

This study aims to pilot LUS in Mozambique and Pakistan and to generate evidence regarding the use of LUS as a diagnostic tool for childhood pneumonia. Children with cough <14 days with chest indrawing (n=230) and without chest indrawing (n=40) are enrolled. World Health Organization Integrated Management of Childhood Illness assessment is performed at enrolment, along with a chest radiograph and LUS examination. Respiratory and blood specimens are collected for viral and bacterial testing and biomarker assessment. Enrolled children are followed for 14 days (in person) and 30 days (phone call) post-enrolment with LUS examinations performed on Days 2, 6 and 14. Qualitative and quantitative data are also collected to assess feasibility, usability and acceptability of LUS among healthcare providers and caregivers. The primary outcome is LUS findings at enrolment with secondary outcomes including patient outcomes, repeat LUS findings, viral and bacterial test results, and patient status after 14 and 30 days of follow-up.

**Ethics and dissemination:**

This trial was approved by the Western Institutional Review Board as well as local ethics review committees at each site. We plan to disseminate study results in peer-reviewed journals and international conferences.

**Trial registration number:**

NCT03187067.

**Key messages**

- In this study, non-physician healthcare personnel perform and interpret lung ultrasound (LUS) examinations, facilitating an assessment of the appropriate level of healthcare provider for these tasks.
- Serial LUS examinations allow for an assessment of how LUS may be used to characterise the progression of chest indrawing pneumonia in children and may help prioritise which children require further care.
- The study is enrolling in two very different settings with very different study populations, which may reflect distinct underlying pneumonia epidemiologies or potentially lead to difficulties in harmonising the data between sites; however, the ability to assess LUS in two diverse geographies is also a strength of this study.

## Introduction

Pneumonia is the leading infectious cause of childhood mortality worldwide.^1 2^ In addition to preventing pneumonia, there is a critical need to provide greater access to appropriate diagnostics and effective treatment. Childhood pneumonia is challenging to diagnose. Currently, in low-resource settings (LRS), pneumonia is diagnosed using WHO Integrated Management of Childhood Illness (IMCI) guidelines that rely on assessing variable and subjective clinical signs like respiratory rate and chest indrawing.^3^ Given the limitations of these non-specific clinical signs, it is not fully understood how effective WHO IMCI guidelines are in identifying pneumonia.

Diagnostic alternatives to WHO IMCI have challenges as well.^4^ Clinical diagnosis not using WHO IMCI guidelines lacks standardisation and can lead to empiric antibiotic administration. Chest radiography (CXR) can be costly, difficult to obtain and time consuming and exposes the child to ionising radiation.^4 5^ Microbiology (eg, blood culture, lung/pleural aspiration and/or bronchoalveolar lavage) can be slow, is invasive and only detects a limited proportion of cases.^4^ Biomarkers such as C reactive protein can correlate with bacterial infection but have no set threshold and do not indicate a specific aetiology.^4^

Lung ultrasound (LUS) is a promising pneumonia diagnostic technology that has demonstrated high diagnostic accuracy.^5^ There is compelling evidence that indicates that LUS may have greater sensitivity, similar specificity and better interoperator reliability when compared with CXR, a diagnostic not readily available in LRS.^6–10^ Additional advantages of LUS, relative to CXR, include its portability, ease of use, lower cost and absence of ionising radiation.^5 11^ The goal of this pilot is to generate evidence to build a greater consensus regarding the use of LUS as a tool for the diagnosis of childhood pneumonia in LRS.

## Methods and analysis

### Study design and setting

**Figure 1:**
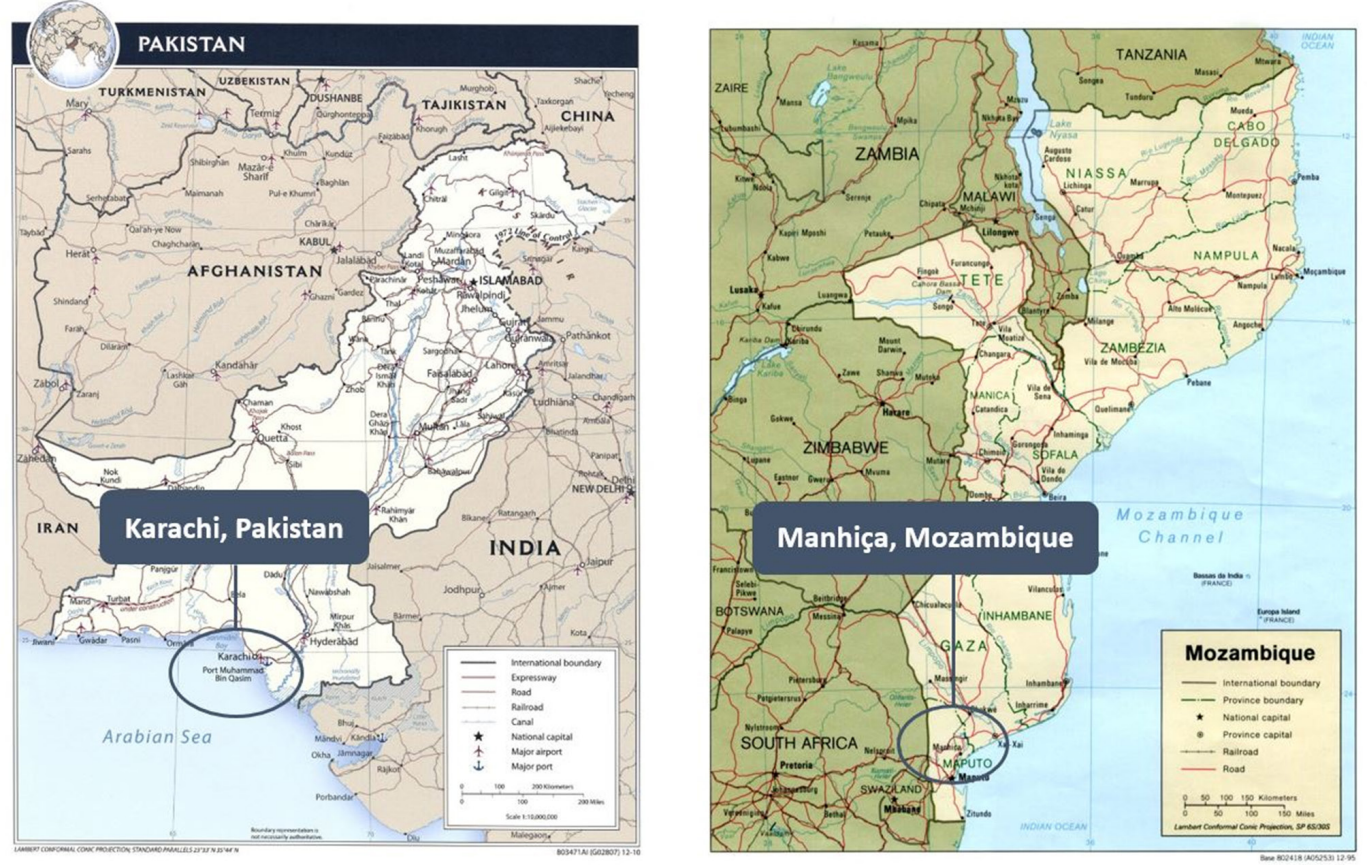
Map of study areas.^16 17^

The primary objectives of this prospective, observational, facility-based cohort study are: (1) to provide scientific evidence assessing whether the addition of LUS to the current pneumonia pathways improves identification of pneumonia in children 2 through 23 months of age presenting to district hospitals in Manhiça, Mozambique and Karachi, Pakistan and (2) to determine whether LUS is feasible, usable and acceptable among healthcare providers (HCPs) and caregivers of children with respiratory symptoms for diagnosing and managing pneumonia in these settings. This study is conducted at Manhiça District Hospital, the referral health facility for the entire Manhiça District in Mozambique and at Sindh Government Children’s Hospital–Poverty Eradication Initiative, a district hospital for the District Central, the largest district in Karachi, Pakistan (figure 1).

### Study participants

Children aged 2 through 23 months presenting to a study hospital with a history of cough with a duration of less than 14 days and/or difficulty breathing are recruited by good clinical practice (GCP)-trained hospital staff during routine intake and screening procedures in the hospitals’ outpatient and/or emergency departments and referred to trained study staff for study screening. To avoid potential selection bias, referred children are screened for enrolment in a sequential manner as much as possible. Referred children are considered for enrolment as cases if they present with chest indrawing or for enrolment as controls if they present with no chest indrawing, fast breathing or fever. Trained study staff assess the child for all inclusion and exclusion criteria (table 1). Final eligibility determination depends on the results of the medical history, clinical examination including WHO IMCI assessment, appropriate understanding of the study and completion of the written informed consent process. Cases and controls may be inpatient or outpatient at the discretion of the study site.

**Table 1:** Study definitions and eligibility criteria

Definitions	
Fast breathing for age	Children 2 to <12 months of age: RR ≥50 breaths per minute Children≥12 months of age: RR ≥40 breaths per minute
Severe respiratory distress	Grunting, nasal flaring and/or head nodding
WHO IMCI general danger signs	Lethargy or unconsciousness, convulsions, vomiting everything, inability to drink or breastfeed
Eligibility criteria	
Inclusion criteria	Cases 2 through 23 months of age Cough <14 days or difficulty breathing Visible indrawing of the chest wall with or without fast breathing for age Ability and willingness of child’s caregiver to provide informed consent and to be available for follow-up for the planned duration of the study, including accepting a home visit if he/she fails to return for a scheduled study follow-up visit Controls 2 through 23 months of age Cough <14 days or difficulty breathing Ability and willingness of child’s caregiver to provide informed consent and to be available for follow-up for the planned duration of the study, including accepting a home visit if he/she fails to return for a scheduled study follow-up visit
Exclusion criteria	Cases Resolution of chest indrawing after bronchodilator challenge, if wheezing at screening examination Severe respiratory distress Arterial SpO_2_ <90% in room air, as assessed non-invasively by a pulse oximeter WHO IMCI general danger signs Stridor when calm Known or possible tuberculosis (history of a cough ≥14 days) Any medical or psychosocial condition or circumstance that, in the opinion of the investigators, would interfere with the conduct of the study or for which study participation might jeopardise the child’s health Living outside the study catchment area Controls Axillary temperature ≥38°C Fast breathing for age Visible indrawing of the chest wall SpO_2_ <95% in room air, as assessed non-invasively by a pulse oximeter WHO IMCI general danger signs Stridor when calm Known or possible tuberculosis (history of a cough ≥14 days) Any medical or psychosocial condition or circumstance that, in the opinion of the investigators, would interfere with the conduct of the study or for which study participation might jeopardise the child’s health Living outside the study catchment area

IMCI, Integrated Management of Childhood Illnesses; RR, respiratory rate; SpO_2_, oxyhaemoglobin saturation.

For the feasibility, usability and acceptability assessment, study HCPs, healthcare administrators and caregivers at each site are invited to participate. HCPs and healthcare administrators are enrolled if they are 18 years or older, involved in or aware of the study and have provided written informed consent. Caregivers are enrolled if they are 18 years or older, have a child enrolled in the study and are willing to participate in an in-depth interview (IDI) as well as direct observation while LUS is performed on their enrolled child.

### Study procedures

Study procedures are conducted according to the most recently approved version of the protocol (current version 1.9, 17 January 2018 (online supplementary appendix 1)). On Day 1, following screening and written informed consent, study staff perform the following procedures: assign participant identification number; collect information on location of residence, sociodemographic characteristics, environmental exposures, vaccination history, concomitant medications and antibiotic use, current illness, pneumonia hospitalisation(s) and medical history; perform a physical examination; obtain CXR and LUS (within 8 hours after physical examination) and collect a respiratory specimen (cases only) and a blood sample.

10.1136/bmjresp-2018-000340.supp1 Supplementary data

Enrolled children are followed through hospital outcome as well as 14 days (in-person either in the hospital, study clinic or the child’s home) and 30 days (phone call) postenrolment (table 2). Caregivers are scheduled to bring their children for repeat LUS examinations and follow-up visits with the study staff on Days 2, 6 and 14. The protocol-specified window for completing each study visit is +24 hours (Days 2 and 6) and ±72 hours (Days 14 and 30). If a child presents to a study hospital during the period of his/her participation, an unscheduled visit may be performed. During follow-up and unscheduled visits, study staff obtain a LUS examination, review any changes in medical history and perform a physical examination. The Day 30 phone call is intended to determine the child’s health status. Should the phone call detect any ongoing health problem, the child is referred to further care. All enrolled children receive local standard of care. Under no circumstances does the participation in the study interfere with or unnecessarily delay the management of sick children.

**Table 2:** Schedule of study visits and evaluations

Activity	Day 1: Screening and enrolment	Day 2*	Day 6*	Day 14†	Day 30 (phone)†	Unscheduled visit
Assess eligibility	✓					
Obtain informed consent	✓					
Assign participant identification number	✓					
Collect/update locator information	✓	✓	✓	✓		✓
Collect sociodemographic information	✓					
Collect information on environmental exposures	✓					
Collect vaccination history	✓					
Collect/update medical history	✓	✓	✓	✓	✓	✓
Assess for general and respiratory danger signs	✓	✓	✓	✓		✓
Respondent assessment of current symptoms	✓	✓	✓	✓	✓	✓
Clinician assessment of current symptoms	✓	✓	✓	✓		✓
Collect vital signs	✓	✓	✓	✓		✓
Perform targeted physical examination	✓	✓	✓	✓		✓
Collect/update concomitant medications/antibiotics	✓	✓	✓	✓	✓	✓
Collect/update hospitalisation log		✓	✓	✓	✓	✓
Perform chest X-ray	✓					
Perform lung ultrasound	✓	✓	✓	✓		✓
Collect respiratory specimens	✓					
Collect blood sample	✓					
Refer to clinical care (as needed)	✓	✓	✓	✓	✓	✓
Schedule next visit	✓	✓	✓	✓		
End of study questions					✓	

*Window: +24 hours.

†Window: +/-72 hours days.

### Lung ultrasound and chest radiography examinations

LUS examinations are performed by trained non-physician healthcare personnel who received a 3-day standardised training course as well as 2 days of supervised practice prior to the initiation of study activities. During the LUS examination, longitudinal and oblique scans are obtained of the anterior, lateral and posterior sides of the child’s chest. Six areas are examined on each enrolled child, comprising the anterior, lateral and posterior areas of the lungs, further divided into the upper and lower halves. Study personnel begins by conducting a longitudinal scan in each of the aforementioned areas. If any abnormality (eg, consolidation) is identified, an oblique scan is performed. The child is examined in his/her most comfortable position (eg, the caregiver’s arms).

To ensure quality and consistency of LUS collection and interpretation, on-site LUS training is performed prior to study initiation, video refresher trainings are performed as necessary during the study, and a LUS standard operating procedures (SOP) document provides an a priori description of features typical to pneumonia diagnosis. A standardised LUS reporting form includes all chest areas examined as well as the patterns detected (ie, interstitial syndrome, consolidation, air bronchogram and pleural effusion; online supplementary appendix 2).^8^ The LUS interpretation targets the detection of typical subpleural lung consolidations with tissue-like or anechoic patterns and blurred, irregular margins. LUS is considered positive when consolidation and/or pleural showing features described in the LUS SOP is present on imaging. At least two independent physicians trained in LUS interpret each examination. If discordant, a designated LUS interpretation expert acts as a tiebreaker. In addition to the aforementioned interpretation, the LUS operators at each site also interpret each examination in order to facilitate comparison between interpretations by physicians with extensive LUS training and those done by newly trained study personnel. To minimise bias, on-site LUS interpretation is performed after the fact (rather than in real-time) in batches, including examinations from at least four children. Results from the study LUS examinations do not inform or impact the child’s clinical care.

10.1136/bmjresp-2018-000340.supp2 Supplementary data

CXR images are collected based on the current standard practice at each study site. The CXR interpretation panel investigates radiographic indicators of primary end-point pneumonia, in a process modelled after the WHO CXR standardised interpretation process.^12–14^ Interpretation focuses on the presence of consolidation, infiltrates and/or effusion as well as additional findings (eg, atelectasis) to facilitate additional comparisons with LUS.^8^ At least two independent CXR-trained physicians interpret each CXR. If discordant, a designated CXR expert acts as tiebreaker. CXR collection and interpretation is further detailed in a CXR SOP.

### Sample collection and testing

For all cases, a blood sample is collected for haemoglobin, malaria (Mozambique only), HIV screening (Mozambique only), bacterial invasive disease screening and biomarker assessment. For controls, a finger stick or blood sample for haemoglobin, malaria (Mozambique only), HIV screening (Mozambique only) is collected. Respiratory samples are collected from all cases. Nasopharyngeal swabs (Pakistan) or aspirates (Mozambique) are collected for respiratory viral and bacterial PCR using a commercial multiplex test. In Mozambique, nasopharyngeal swabs are also collected to evaluate carriage of bacterial pathogens.

### Qualitative substudy

A mixed methods evaluation is conducted to assess the feasibility, usability and acceptability of LUS for diagnosing childhood pneumonia in a LRS. Semistructured IDIs are conducted among HCPs and administrators associated with the study. Direct observation and IDIs are conducted among caregivers of children enrolled in the overall study. During the IDIs, trained qualitative study staff introduce each question separately. If study staff do not get a satisfactory response (eg, the respondent does not understand the question, the respondent does not provide information that answers the question, the response lacks detail and so on), study staff repeat the question and probe for additional information, but do not prompt any responses. The IDIs are conducted in the language best understood by the caregiver (ie, Portuguese or Changana in Mozambique; Urdu or Sindhi in Pakistan). During the direct observation, study staff record how much time was spent explaining the LUS examination to the caregiver as well as any questions, comments or reactions by the caregiver.

### Sample size

The total sample size for this study is 270 children (100 cases and 20 controls at the Mozambique site; 130 cases and 20 controls at the Pakistan site). This is a pilot study seeking to demonstrate feasibility of investigating LUS for pneumonia diagnosis and prognosis in a district facility setting. Its underlying purpose is to generate evidence to inform future full-scale studies. Thus, the sample size may not provide adequate power to answer all of the research questions laid out in this protocol. The sample size was selected to maximise the amount of information collected within the confines of the available resources. With the proposed 230 case participants, if an estimated 30%–40% of enrolled children with chest indrawing is found to have pneumonia by CXR, we would have a sample of 70–90 case participants with radiologically confirmed pneumonia. We believe that this is sufficient to generate evidence and inform future studies regarding the use of LUS as a tool for pneumonia diagnosis and prognosis.

For the feasibility, usability and acceptability assessment, the total sample size is approximately 20 participants in each cohort (10 HCPs/administrators and 10 caregivers at each site). All study HCPs are invited to participate in the data collection procedures for this portion of the project; healthcare administrators may also be invited to participate.

### Data collection and quality assurance

Quantitative study data are collected by clinical study staff using designated source documents or paper-based case report forms which are then entered into a REDCap electronic database or via direct data entry into REDCap.^15^ Qualitative study data are collected using paper-based forms and audio recordings which are subsequently transcribed for analysis.

Clinical research data are maintained through a combination of secure electronic data management system and physical files with restricted access to ensure confidentiality. Two distinct study databases are maintained separately by each study site: the primary study database with study visit data and a database with participating children’s personally identifiable information. To ensure accuracy and completeness, data are routinely reviewed by the sponsor through quality assurance reviews, audits and evaluation of the study safety and progress. Standard GCP is followed to ensure accurate, reliable and consistent data collection.

### Data management

Primary data management activities, which include data entry and validation, data cleaning, database quality control and disaster recovery plans are undertaken at each study site and are overseen by the on-site data manager. Data review, oversight and preparation of final study database are performed by the sponsor in collaboration with the study sites. Data are maintained in databases hosted at each study site. All data management activities are in compliance with International Council on Harmonization (ICH) GCP E6, sponsor organisation and institutional requirements for the protection of children and confidentiality of personal and health information.

### Outcomes

We hypothesise (stated under the alternative) that for the diagnosis of CXR-confirmed pneumonia (radiological endpoint pneumonia), the specificity of the WHO IMCI clinical assessment algorithm plus LUS is greater than the specificity of the WHO IMCI clinical assessment algorithm alone. The primary endpoint is the proportion of children with pneumonia suggested by LUS and/or CXR on enrolment. Secondary endpoints include the proportion of children with pneumonia suggested by CXR but not LUS or pneumonia suggested by LUS but not CXR. A full list of endpoints is presented in box 1. We also are conducting exploratory investigations regarding whether LUS may be able to help characterise and prioritise which children require hospitalisation or are at higher risk of progression of their pneumonia or acute process, whether LUS can identify characteristic imaging differences in viral versus bacterial versus mixed pneumonia and whether LUS is feasible, usable and acceptable among HCPs and caregivers for diagnosing paediatric pneumonia in a LRS.

Box 1 **: Study endpoints**

Primary endpoint

- Proportion of children with pneumonia suggested by LUS and/or CXR.

Secondary endpoints

- Proportion of children with pneumonia suggested by CXR but not LUS.
- Proportion of children with pneumonia suggested by LUS but not CXR.
- Proportion of children with no pneumonia identified.
- Aetiological diagnosis for each recruited child with samples collected.
- Proportion of children with a positive viral PCR.
- Difference in characteristic LUS imaging patterns between children with viral vs bacterial versus mixed pneumonia.
- Clinical and/or diagnostic biomarker outcomes.
- Patient status after 6 days of follow-up.
- Patient status after 14 days of follow-up.
- Patient status after 30 days of follow-up.
- Feasibility, usability and acceptability of LUS among HCPs.
- Acceptability of LUS among caregivers.

CXR, chest radiography; HCP, healthcare provider; LUS, lung ultrasound.

### Statistical analyses

Sensitivity and specificity are calculated and McNemar’s test of paired data is used to compare discordance in results between LUS examination and the WHO IMCI clinical assessment algorithm. This is performed as a two-sided test with alpha=0.05. Interrater agreement for CXR and LUS is determined using kappa statistics.

Feasibility, usability and acceptability are assessed through qualitative data analysis of IDIs with HCPs and caregivers as well as direct observation with caregivers. The qualitative data are in narrative format and the results are descriptive. The transcripts are coded and analysed using a codebook and themes identified a priori, including opportunities and barriers for introduction of LUS, feasibility of implementing LUS and perceived value. Qualitative data analysis software (NVivo) is used to organise, code and analyse the qualitative data in an iterative process.

## Ethics and dissemination

### Ethical approvals and consent

The study is conducted in accordance with the ICH GCP and the Declaration of Helsinki 2008. Written informed consent is obtained in the local language by trained study staff from all eligible children’s caregivers prior to enrolment.

### Possible risks

Caregivers may feel compelled to enrol in the study in order to receive care for their child within a research setting, which may be perceived as of a higher quality than the standard of care. In order to minimise the risk of coercion, hospital staff inform caregivers about the study and refer only those who are interested. During the informed consent process, study staff emphasise that the child will receive the required medical care whether enrolled in the study or not. Other potential risks to study participation may include those associated with collection of respiratory and blood specimens, CXR and LUS examinations and delayed medical management. In order to minimise the discomfort and risks associated with respiratory and blood specimen collection, study staff collecting specimens from children are trained in the appropriate procedures and supervised accordingly. Whenever possible, research blood draws are combined with clinical blood draws to minimise the amount of needle sticks experienced by the child. Study staff implementing CXR are trained in appropriate procedures and supervised accordingly with standard precautions in place to protect children from ionising radiation. To minimise the dose of ionising radiation, only one CXR is obtained during the study period; there are no repeat CXR assessments unless clinically indicated. Study staff implementing LUS examinations are trained in appropriate procedures and supervised accordingly with standard precautions in place. In order to minimise the possibility that participation in this study interferes with the medical management of hospitalised children, study staff are trained in integrating research procedures with clinical care. Urgent clinical care for acute medical issues is always prioritised above research procedures. Extreme care is taken to ensure that no necessary treatment is delayed to accommodate study procedures.

### Dissemination

We plan to disseminate study results in peer-reviewed journals and international conferences, targeting those involved in the clinical care of children in LRS as well as those who develop and advise on policies and guidelines in those settings. The trial is registered with ClinicalTrials.gov (registration number NCT03187067).

### Efforts towards generalisable results

The study was carefully developed and pragmatically designed with inclusion and exclusion criteria to allow for the most generalisable results possible without putting children with danger signs or severe respiratory distress at risk. Children with comorbidities such as malnutrition, anaemia, HIV and malaria are included in order to enrol a broad group of children. Enrolled cases have been diagnosed with chest indrawing pneumonia based on WHO IMCI clinical guidance, as is common in LRS where children are typically diagnosed based on clinical criteria alone.

### Efforts towards rigorous protocol

Dedicated study staff trained in GCP, IMCI and study-specific procedures follow children enrolled in the trial to assure the protocol and SOPs are followed and data are accurately collected. Standardised training, supervision and oversight are undertaken to ensure quality, consistency and harmonised trial procedures and implementation. Regular monitoring is provided by Save the Children to assess compliance with human subjects and other research regulations and guidelines, adherence to the study protocol and procedures and quality and accuracy of data collected.

### Limitations and bias

Limitations to this study and potential sources of bias include the sampling strategy, harmonising the data between study sites, loss to follow-up and LUS and/or CXR interpretation bias. As noted above, given that the study is a pilot, the sample size may not provide adequate power to answer all of the proposed research questions. Because the study sites and the populations the hospital facilities serve are so different from each other, and the sample sizes of the enrolled children are relatively small at each study site, it may be difficult to harmonise the data between sites or data may simply represent the different underlying pneumonia epidemiologies. To mitigate this risk, we are applying the same enrolment criteria between study sites and are training study staff at both sites according to the same standardised protocols. We are also monitoring study staff performance with multiple quality control procedures and interim trainings. To minimise loss to follow-up, community sensitisation and outreach are performed and caregivers of enrolled children are provided with clear follow-up instructions and reminders prior to the follow-up visits. Home visits take place among children who missed a follow-up visit. To eliminate LUS and/or CXR interpretation bias, trained panels of CXR and LUS experts interpret all images. The panels are blinded to each other’s interpretations, and discordant interpretations are adjudicated in a process modelled after the WHO CXR process.^12^ LUS and CXR interpretations are based on a priori guidance set forth in the LUS and CXR SOPs.
