# Supplementary material for: Multicentre pilot study evaluation of lung ultrasound for the management of paediatric pneumonia in low-resource settings: a study protocol
Source: BMJ Open Respir Res. 2018 Dec 19;5(1):e000340. doi: 10.1136/bmjresp-2018-000340 (PMC6307622; doi:10.1136/bmjresp-2018-000340)
Supplement: Supplementary data [file bmjresp-2018-000340supp001.pdf]

Pediatric Pneumonia Lung Ultrasound (PLUS)

Full title: Evaluation of impact of lung ultrasound (LUS) on management of pneumonia in low-resource settings, and feasibility, usability and acceptability of this technology: A pilot study

A Study of the Pneumonia Innovations Team
Sponsored by:
Save the Children Federation, Inc., United States

Version 1.9
17 January 2018

Principal Investigator:
Amy Ginsburg, MD, MPH, Save the Children Federation, Inc., United States

Investigators, Mozambique site	Investigators, Pakistan site
**Local Principal Investigator:** Dr. Quique Bassat (1)(2)<br>**Co-Investigators:** Dr. Rubao Joao Bila (1); Dr. Antonio Sitoe (1); Dr Sozinho Acácio (1); Dr. Rosauro Varo (1)(2); Dr. Lola Madrid (1)(2); Dr. Betuel Sigaúque (1) Dr. Carmen Muñoz Almagro (3)	**Local Principal Investigator:** Dr. Fyezah Jehan (4)<br>**Co-Investigators:** Dr. Imran Nisar (4); Dr. Naila Nadeem (4); Dr. Neel Kanth (5)

(1) Centro de Investigação em Saúde da Manhiça (CISM)
Manhiça; CP 1929 Maputo, Moçambique
(2) Barcelona Institute for Global Health (ISGlobal)
Rosselló 132, 08036; Barcelona, Spain
(3) Pediatric Infectious Diseases Unit, Pediatrics Department, Hospital Sant Joan de Déu (University of Barcelona), Barcelona, Spain. Passeig Sant Joan de Déu 2, 08950, Esplugues de Llobregat (Barcelona – Spain
(4) Aga Khan University, Pakistan
Stadium Rd, Karachi 74800, Pakistan
(5) Sindh Government Children's Hospital – Poverty Eradication Initiative
North Karachi, Pakistan

Confidentiality Statement

This document is confidential and is to be distributed for review only to investigators, potential investigators, consultants, PLUS study staff, applicable regulatory authorities, and applicable independent ethics committees or institutional review boards. The contents of this document shall not be disclosed to others without written authorization from Save the Children (or others, as applicable), unless it is necessary to obtain informed consent from potential PLUS study participants.

Pediatric Pneumonia Lung Ultrasound (PLUS)

TABLE OF CONTENTS

TABLE OF CONTENTS 2
ABBREVIATIONS AND ACRONYMS 4
INVESTIGATORS 5
PARTICIPATING INSTITUTIONS 6
EXECUTIVE SUMMARY 7
PROTOCOL OUTLINE 9
1 BACKGROUND AND INTRODUCTION 11
2 RATIONALE 14
3 STUDY HYPOTHESIS, OBJECTIVES AND ENDPOINTS 15
3.1 Study Hypothesis 15
3.2 Study Objectives 15
3.3 Study Endpoints 15
4 METHODOLOGY 16
4.1 Study Design 16
4.2 Study Site 17
4.3 Study Population 17
4.3.1 Study Population Overview 17
4.3.2 Participant Eligibility 18
4.3.3 Sample Size 20
4.4 Study Period 20
5 STUDY PROCEDURES 21
5.1 Recruitment 21
5.2 Screening 21
5.3 Informed Consent Process 22
5.4 Enrollment Visit 22
5.5 Follow-Up Visits 23
5.6 Unscheduled Visits 24
5.7 Missed Visits 24
5.8 Withdrawal and Early Termination 25
5.9 Study Termination Visit 25
5.10 Participant Reimbursement 25
6 RADIOLOGY AND SPECIMENS 26
6.1 LUS Collection and Interpretation 26
6.2 CXR Collection and Examination 27
6.3 Specimen Collection 27
6.4 Specimen Transport 28
6.5 Specimen Processing and Testing 28
7 DATA COLLECTION 28

7.1 Case Report Forms 28
7.2 Source Documents 29
7.3 Data Management 29
7.4 Data Access 29
7.5 Data Storage 29
8 TRAINING REQUIREMENTS, SAFETY ASSESSMENTS AND REPORTING 30
8.1 Training Requirements 30
8.2 Monitoring 30
8.3 Study Discontinuation 31
9 STATISTICAL DESIGN AND ANALYSIS 31
9.1 Overview and General Design 31
9.2 Hypothesis 31
9.3 Analytical Methodology 32
9.4 Result Presentation 32
9.5 Dissemination of Results 32
10 ETHICAL CONSIDERATIONS AND CONSENT 32
10.1 Principles for Clinical Research 32
10.2 Institutional Review Boards (IRBs) and Independent Ethics Committees (IECs) 33
10.3 Informed Consent Documentation 33
10.4 Risks and Benefits 33
10.4.1 Risks to Participants 33
10.4.2 Protection against Risks 34
10.4.3 Benefits to Participants 35
10.5 Participant Confidentiality 35
10.6 Biohazard Containment 35
10.7 Storage of Specimens 35
11 POSSIBLE CONSTRAINTS 36
12 STUDY CHRONOGRAM 38
APPENDICES 39
Appendix I: Study Flow Diagram 39
Appendix II: Schedule of Study Visits and Evaluations 40
Appendix III: Specific Information Regarding the Manhiça site 41
PROTOCOL REFERENCES 54

ABBREVIATIONS AND ACRONYMS

AIDS	Acquired Immunodeficiency Syndrome
AKU-P	Aga Khan University, Pakistan
AUC	area under curve
CDC	Centers for Disease Control and Prevention
CISM	Centro Investigação em Saúde de Manhiça
CRF	case report form
CT	computed tomography
CXR	chest radiography
GCP	good clinical practice
GNI	gross national income
HCP	healthcare provider
HDI	Human Development Index
ICF	informed consent form
ID	identification
IDI	in-depth interview
IEC	Independent Ethics Committee
IMCI	Integrated Management of Childhood Illness
IRB	Institutional Review Board
ISGlobal	Barcelona Institute for Global Health
km	kilometer(s)
LAR	legally authorized representative
LRS	low-resource settings
LUS	lung ultrasound
MUAC	mid upper arm circumference
NP	nasopharyngeal
NPA	nasopharyngeal aspirate
PI	principal investigator
PLUS	Pediatric Pneumonia Lung Ultrasound
QC	quality control
ROC	receiver operating characteristic
SAM	severe acute malnutrition
SCI	Save the Children International
SGCH	Sindh Government Children's Hospital
SC-US	Save the Children Federation Inc., United States
SLGU	San Luigi Gonzaga University
SOP	standard operating procedure(s)
TB	tuberculosis
WHO	World Health Organization
WINFOCUS	World Interactive Network Focusing on Critical Ultrasound
WIRB	Western Institutional Review Board

Pediatric Pneumonia Lung Ultrasound

INVESTIGATORS

Principal Investigator (PI):
Amy Ginsburg, MD, MPH
Senior Advisor, Child Health
Save the Children Federation, Inc., United States

____
PI signature Date

Co-Investigators:
Giovanni Volpicelli, MD, FCCP
Emergency Physician, Department of Emergency Medicine
San Luigi Gonzaga University Hospital

Fyezah Jehan, MBBS, MSc
Assistant Professor, Pediatrics and Pediatric Infectious Diseases
Aga Khan University, Pakistan

Quique Bassat, MD, MSc, PhD
ICREA Research Professor
Barcelona Institute for Global Health
and
Centro de Investigação em Saúde de Manhiça

PARTICIPATING INSTITUTIONS

**Study Oversight/ Management:**	Save the Children Federation, Inc., United States<br>501 Kings Highway East, Suite 400<br>Fairfield, CT 06825
**Study Sites:**	Mozambique: Centro de Investigação em Saúde de Manhiça (CISM; Manhiça Heath Research Centre)/Fundação Manhiça (Manhiça Foundation)<br>Pakistan: Sindh Government Hospital (SGCH), Karachi
**Study Operations:**	Save the Children Federation, Inc., United States<br>Save the Children International, Mozambique<br>Centro Investigação em Saúde de Manhiça<br>Barcelona Institute for Global Health<br>Aga Khan University, Pakistan
**Local Collaborators:**	Centro Investigação em Saúde de Manhiça, Mozambique<br>Aga Khan University, Pakistan
**Technical Support**	San Luigi Gonzaga University
**Funding Agency**	The Bill & Melinda Gates Foundation<br>500 Fifth Avenue North<br>Seattle, WA 98109<br><br>Save the Children Federation, Inc., United States<br>501 Kings Highway East, Suite 400<br>Fairfield, CT 06825

EXECUTIVE SUMMARY

Pneumonia continues to be the leading infectious cause of death in children worldwide. Despite the roll-out of immunizations to protect against *Streptococcus pneumonia* and *Haemophilus influenzae*, mortality from pneumonia remains high. To address this persistent mortality, it is critical to explore modalities that can improve outcomes. Childhood pneumonia is difficult to diagnose. Currently, in low-resource settings (LRS), pneumonia is diagnosed using World Health Organization (WHO) Integrated Management of Childhood Illness (IMCI) guidelines that rely on assessing variable and subjective clinical signs like respiratory rate and chest indrawing. Given the limitations of these clinical signs, it is not fully understood how effective WHO IMCI guidelines are in identifying pneumonia.

Lung ultrasound (LUS) is a promising pneumonia diagnostic technology. There is compelling evidence that indicates that LUS may be as sensitive and specific, and may have greater inter-operator reliability when compared to chest radiography (CXR), a diagnostic not readily available in lower-level inpatient facilities in developing countries. Additional advantages of LUS, relative to CXR, include its portability, ease of use, lower cost, and absence of ionizing radiation. There is great potential for the use of LUS in LRS where there are few, if any, diagnostics for pneumonia, yet high patient volumes and abundant need.

The goal of this project is to generate evidence to build a greater consensus regarding the use of LUS as a tool for the diagnosis of childhood pneumonia in developing countries. A consortium of Save the Children Federation Inc., United States (SC-US), San Luigi Gonzaga University (SLGU), Centro de Investigação em Saúde de Manhiça (CISM) in collaboration with the Barcelona Institute for Global Health (ISGlobal) and Aga Khan University, Pakistan (AKU-P) will conduct a prospective observational cohort study to assess the utility of point-of-care LUS to diagnose childhood pneumonia in Manhiça, Mozambique and Karachi, Pakistan. The primary outcome is to provide scientific evidence assessing whether the addition of LUS to the current pneumonia care pathways in these countries will improve identification of pneumonia in children 2 through 23 months of age presenting to sub-district hospitals, and to help answer the fundamental question of how effective are the WHO IMCI guidelines in identifying pneumonia when LUS is utilized as the reference standard?

To determine how effective the WHO IMCI guidelines are in identifying pneumonia, we will conduct an observational study among 270 children aged 2 through 23 months presenting to district hospitals in Mozambique and Pakistan (100 cases and 20 controls in Mozambique; 130 cases and 20 controls in Pakistan). Eligible cases will present with cough <14 days and/or difficult breathing and chest indrawing. Eligible controls will present with cough <14 days and/or difficult breathing with no chest indrawing, fast breathing or fever. Cases and controls may have comorbidities including HIV infection or exposure, severe acute malnutrition, malaria, or severe anemia. Children will receive local standard of care including WHO IMCI assessment and management as well as CXR and LUS performed at enrollment. Expert physician panels will

interpret the CXR and LUS to ensure consistency and accuracy of interpretation. Respiratory specimens for viral and bacterial testing may be collected from children at enrollment, along with blood for disease screening. Enrolled children will be followed through hospital outcome, as well as 14 days (in person) and 30 days (phone call) post-enrollment. LUS will be performed on enrollment, and on days 2, 6, and 14. The primary outcome will be LUS findings at enrollment with secondary outcomes including patient outcomes, repeat LUS findings, viral and bacterial test results, and patient status after 14 and 30 days of follow-up. Qualitative and quantitative data will also be collected to assess feasibility, usability and acceptability among healthcare providers (HCPs) and caregivers.

In addition to generating evidence regarding the value of LUS in identification of pneumonia, these data will also help to assess whether LUS may be able to help characterize and prioritize which children require hospitalization or are at higher risk for progression. Thus, further secondary aims include using LUS as a prognostic tool to track the progression of pneumonia and to predict disease severity. Finally, the collection of respiratory specimens will allow for the investigation of whether LUS may be able to identify a difference in characteristic imaging patterns between viral, bacterial and mixed pneumonia, creating the potential for improved pneumonia management and a reduction in unnecessary antibiotic use.

By generating this evidence to use and accelerate a biomedical imaging technology such as LUS as a point-of-care device, a quantum paradigm shift and advance over present-day approaches to the detection and diagnosis of pneumonia can be achieved in LRS. LUS has the potential to revolutionize the ability to rapidly and accurately diagnose pneumonia in children, even in LRS, and to ensure that treatment is provided efficiently and rationally, leading to significant impact on childhood mortality.

The following protocol describes activities related to this multicenter clinical study to be conducted in Mozambique (Manhiça) and Pakistan.

PROTOCOL OUTLINE

**Title:**	**Pediatric Pneumonia Lung Ultrasound (PLUS)**: Evaluation of impact of lung ultrasound (LUS) on management of pneumonia in low-resource settings, and feasibility, usability and acceptability of this technology
**Study Oversight and Management:**	Save the Children Federation Inc., United States
**Collaborating Organizations:**	Save the Children Federation Inc., United States<br>Save the Children International, Mozambique Country Office<br>Barcelona Institute for Global Health<br>Centro de Investigação em Saúde de Manhiça/Fundação Manhiça<br>Aga Khan University, Pakistan<br>San Luigi Gonzaga University
**Funding Sources:**	Bill & Melinda Gates Foundation<br>Save the Children Federation Inc., United States
**Rationale:**	To evaluate the impact of LUS on the diagnosis and management of childhood pneumonia in developing countries
**Population:**	Mozambique:<br>• 100 cases aged 2 through 23 months with cough <14 days and/or difficulty breathing **and chest indrawing;**<br>• 20 controls aged 2 through 23 months with cough <14 days and/or difficulty breathing **but no chest indrawing, fast breathing or fever.**<br>Pakistan:<br>• 130 cases aged 2 through 23 months with cough <14 days and/or difficulty breathing **and chest indrawing;**<br>• 20 controls aged 2 through 23 months with cough <14 days and/or difficulty breathing **but no chest indrawing, fast breathing or fever.**<br>All study HCPs at both sites and up to 24 caregivers at each site will be asked to participate in the feasibility, usability and acceptability assessment.
**Schema:**	Cases and controls will be enrolled at a 5:1 ratio, for a total of 200 cases and 40 controls.

Study Groups	N	Day 1	Day 2	Day 6	Day 14	Day 30*
Mozambique: Cases	100	X	X	X	X	X
Mozambique: Controls	20	X	X	X	X	X
Pakistan: Cases	130	X	X	X	X	X
Pakistan: Controls	20	X	X	X	X	X

*Day 30 assessment completed via phone

**Objectives:**	Primary:<br>• To provide scientific evidence assessing whether the addition of LUS to the current pneumonia pathways in Mozambique and Pakistan improves identification of pneumonia in children 2 through 23 months of age presenting to district hospitals<br>• To determine whether LUS is feasible, usable and acceptable among healthcare providers and caregivers of children with respiratory symptoms for diagnosing and managing pneumonia<br><br>Secondary:<br>• To assess whether the specificity of the WHO IMCI criteria increases when LUS is added to the current diagnostic pathway<br>• To assess whether LUS may be able to help characterize and prioritize which children require hospitalization or are at higher risk for progression of the pneumonia or their acute process<br>• To investigate whether there are different characteristic LUS imaging patterns between viral, bacterial and mixed pneumonia
**Endpoints:**	Primary:<br>Proportion of children with pneumonia suggested by LUS and/or CXR<br><br>Secondary:<br>Proportion of children with pneumonia suggested by CXR but not LUS<br>Proportion of children with pneumonia suggested by LUS but not CXR<br>Proportion of children with no pneumonia identified<br>Etiological diagnosis for each recruited child with samples collected<br>Proportion of children with a positive viral PCR<br>Difference in characteristic LUS imaging patterns between children with viral versus bacterial versus mixed pneumonia<br>Clinical and/or diagnostic biomarker outcomes<br>Patient status after 6 days of follow-up<br>Patient status after 14 days of follow-up<br>Patient status after 30 days of follow-up<br>Feasibility, usability and acceptability of LUS among HCPs<br>Acceptability of LUS among caregivers
**Timeline:**	Projected duration of enrollment is about 12 months.<br>All children will be followed for 14 days (in person) and 30 days (via phone) after enrollment.

Pediatric Pneumonia Lung Ultrasound (PLUS)

1 BACKGROUND AND INTRODUCTION

Pneumonia is the leading cause of infectious death among children less than 5 years of age. Estimates indicate that 935,000 children died globally from pneumonia in 2013, accounting for 15% of all under 5 child deaths.[1] The high burden of childhood pneumonia deaths belies the fact that pneumonia-related mortality is preventable if pneumonia is identified and treated appropriately. Accurate diagnosis and prompt case management are critical elements of pneumonia control strategies. However, identifying pneumonia can be challenging, and pneumonia too often goes unrecognized and can result in death.

Recognizing fast breathing by counting the respiratory rate and identifying chest indrawing is integral to the current practices of diagnosing childhood pneumonia in LRS.[2] However, counting respiratory rate and appreciating chest indrawing is notoriously difficult, and high inter-observer variability is often demonstrated.[3,4] Furthermore, these clinical signs are not sufficiently specific, sensitive, or reliable for diagnosing pneumonia.[5] Misdiagnosis of pneumonia using clinical signs is common, and it is unknown how many deaths result as a consequence. Reliance on clinical examination findings alone also results in children being treated with antibiotics inappropriately, leading to increased risk of antibiotic resistance.[6,7]

Despite it being an imperfect reference standard with a high degree of inter- and intra-observer variability in interpretation, chest radiography (CXR) if available and accessible, is the standard imaging test of choice in well-resourced settings for diagnosing pneumonia in children.[8] Yet, the World Health Organization (WHO) has estimated that as many as three-quarters of the world's population do not have access to diagnostic imaging services.[9] Furthermore, CXR is costly and exposes children to ionizing radiation.[10] Chest computed tomography (CT) has been shown to be a more accurate reference standard than CXR, especially for very small lung consolidations and pleural effusions. However, obtaining chest CT in children with suspected pneumonia is not standard of care given its higher dose of ionizing radiation and increased cost, and general unavailability of CT scanners in LRS.

There is an urgent need for innovations in pneumonia diagnosis to reduce the burden of childhood pneumonia globally, especially as CXR is cost-prohibitive in many LRS with the highest burden. Diagnosis methods must be specific, sensitive, and reliable enough to accurately identify children with pneumonia, but they also need to be affordable enough to be accessible in LRS. Some diagnostics such as biomarker discovery and assay work are in development, but it

is unclear how quickly these may be available and what cost implications they may have in LRS. Meanwhile, other relatively simple diagnostic tools are available now and need to be scaled to reach communities that need them. Accelerating these innovations' impact and creating new ones could transform pneumonia diagnosis and treatment and save the lives of millions of children.

Lung ultrasound (LUS) is an example of one such innovation. Due to recent significant advances in technology making portable or handheld ultrasound machines more accurate and more available, point-of-care ultrasound use has grown.[11-14] Using LUS in the diagnosis of pneumonia has been shown to be both accurate and feasible.[15-26] LUS can facilitate the diagnosis of pneumonia by identifying sonographic air bronchograms (hyperechoic linear elements representing air in bronchioles) within free breath dependent motion of lung consolidation (subpleural hypoechoic or tissue-like region with blurred margins and irregular shapes).[13,15,16,18,26-29] LUS has been found to be better than CXR for identifying bronchiolitis and pleural effusions and can differentiate infiltrates from atelectasis.[15,23,26,30]

In a systematic review and meta-analysis that included ten studies and 1,172 adult patients with suspected pneumonia, pooled sensitivity and specificity for pneumonia diagnosis using LUS, compared to CXR, were high, at 94% (95% CI, 92%–96%) and 96% (95% CI, 94%–97%), respectively.[31] Point-of-care LUS may be particularly useful also in children who have thinner chest walls and smaller lung mass compared with adults.[15,16] As the majority of pediatric pneumonias are subpleural and begin peripherally, they may be visible by LUS.[32] LUS also has been found suitable for diagnosing pneumonia in neonates.[13,33] LUS may be helpful for following disease progression. In a small study following children with pneumonia, the average size of the pneumonia patch decreased over two weeks.[34] An international panel of experts reviewed the literature and developed a series of evidence-based recommendations for point-of-care ultrasound that included the role of LUS as a clinically useful tool in children with suspected pneumonia, noting that it was as accurate as CXR in the diagnosis of childhood pneumonia.[13] LUS can differentiate consolidations due to pneumonia, atelectasis, or pulmonary embolism, and is able to monitor aeration changes and the effects of therapy. In a meta-analysis that included nine studies and 1,080 adults and children patients, LUS performed better than CXR and with high accuracy in diagnosing pneumonia in children.[35] In a 2015 meta-analysis that included eight studies and 765 children with high overall methodologic quality but heterogeneity, LUS demonstrated a sensitivity of 96% (95% CI, 94%–97%) and a specificity of 93% (95% CI, 90%–96%) and positive and negative likelihood ratios were 15.3 (95% CI, 6.6–35.3) and 0.06 (95% CI, 0.03–0.11), respectively. The area under the receiver operating characteristic curve was 0.98. [36]

To date, the only published study in LRS evaluating the use of LUS for diagnosis of childhood pneumonia compared LUS to the WHO clinical diagnosis algorithm in 378 children in Peru and Nepal.[37] This study did not obtain CXR imaging, but using LUS as the reference, the WHO

algorithm had a sensitivity of 70% (95% CI, 56%–81%) and a specificity of 69% (95% CI, 54%–65%). The inter-observer agreement for LUS diagnosis between general practitioners was 0.79 (95% CI, 0.73–0.81). While point-of-care LUS can be effectively used in a variety of clinical settings in LRS to enhance the diagnosis of childhood pneumonia, to date, LUS has not been widely adopted in pediatric care.

Point-of-care LUS could transform health care, obviating the need for more expensive and time-consuming CXR imaging with its attendant ionizing radiation exposure. This novel real-time approach of using LUS to diagnose pneumonia trains HCPs to utilize a standardized lung scanning and image interpretation protocol. For example, the World Interactive Network Focusing on Critical Ultrasound (WINFOCUS) has developed a series of protocols and trainings for point-of-care ultrasound use for a variety of purposes, including LUS diagnoses. WINFOCUS has hosted trainings around the world and is pushing to standardize practices while advocating for expanded use in health facilities.[38] A practice-based ultrasound curriculum and training program can be implemented in LRS. Easy to learn and quick to perform, LUS is less technically demanding than other sonographic examinations.[39,40] Multiple groups have reported that HCPs can be quickly and easily trained in LUS for the diagnosis of pneumonia.[16,37] It is rapid, repeatable, reliable, and independent from specific acoustic windows, and therefore, suitable for a meaningful evaluation in many different settings.[41] LUS examines the periphery of large internal organs, easily accessible by placing the probe on the chest wall.

In addition, LUS is less costly and can be performed in less time than CXR.[37,42-46] CXR requires specialized equipment, personnel, and power, whereas LUS does not require an area protected from radiation, can be operated by non-technicians, and can be easily portable and performed in the absence of an energy supply (using rechargeable batteries). In addition to its potential in diagnosing pneumonia, ultrasound can be used for evaluation and intervention of many other processes, including peripheral intravenous access and bedside echocardiography, among other applications. For example, cost-savings with ultrasound has been demonstrated at the district hospital level in Sudan where its availability reduced expenditure on other radiological diagnostic procedures.[47] The availability of ultrasound may also facilitate more appropriate care-seeking behaviors by encouraging patients to come to a health facility for skilled care instead of utilizing more traditional methods that may lead to delayed care-seeking and poor outcomes.[48]

LUS is a promising diagnostic alternative to CXR and thoracic CT. A dynamic multipurpose tool, ultrasound works well and is clinically useful in extreme, austere, and diverse environments of space, swamp, jungle, mountain, and desert.[49] There is compelling evidence that indicates that LUS may be more sensitive, specific, and have greater inter-operator reliability than CXR. Additional advantages of LUS include its real-time image display, rapid data collection, lower infrastructure requirements, and safety.[14,49] Despite this, LUS is not widely used. The small sample sizes of studies to date comparing LUS to CXR have not yet convinced many practitioners to move from CXR to LUS, so more comprehensive, robust data are still needed. To date, there

is no universal consensus as to how the images should be interpreted in terms of prognostic value and treatment needs. More data are needed from children. Furthermore, there are no studies from resource-limited countries comparing the performance of LUS to CXR for diagnosing childhood pneumonia.

2 RATIONALE

This pilot study will evaluate whether the addition of LUS to the current pneumonia care pathway will improve diagnosis of pneumonia in young children in LRS, and will help answer the fundamental question of how effective are the WHO IMCI guidelines in identifying pneumonia when LUS is utilized as the reference standard. Additionally, LUS will be compared to CXR. This study will be the first African and multi-country study to compare LUS to CXR for diagnosing childhood pneumonia in LRS. With an accurate and reliable way to diagnose pneumonia, a better understanding of the burden of childhood pneumonia can be achieved. Furthermore, LUS may also be able to help differentiate viral versus bacterial versus mixed etiology of pneumonia in children. A prospective case series of patients enrolled during the 2009 H1N1 influenza pandemic showed that LUS may be able to distinguish the radiologic features of viral (subpleural consolidations and/or B-lines or confluent B-lines) from bacterial (lung consolidation with sonographic air bronchograms) pneumonias with high inter-observer agreement (calculated kappa 0.82).[50] Bacterial infection, especially pneumococcal, is associated with CXR-confirmed pneumonia.[51] Further evaluation of etiology differentiation through LUS is a secondary objective of the proposed research. If LUS can be utilized as a prognostic tool to better predict severity, follow progression, and help differentiate viral versus bacterial versus mixed etiology of pneumonia in children, the advantages of LUS will likely surpass that of other existing diagnostics.

If the evidence in support of LUS is compelling, LUS could have a significant impact on decreasing childhood mortality. LUS could be a critical component in diagnosing and managing childhood pneumonia, and it could be lifesaving, having a significant impact on decreasing childhood mortality. In LRS, people generally do not have access to radiography or other diagnostics. This diagnosis gap is especially important in LRS, where clinical deterioration due to pneumonia is often rapid, especially among children with severe illness. Early and accurate recognition of a child with severe pneumonia is critical to reduce unnecessary child deaths. Incorporating LUS into existing clinical care may allow HCPs to better diagnose and treat pneumonia. Additionally, this change in practice may strengthen the ability to more accurately measure the burden of pneumonia.

This pilot study is the anticipated first step in assessing whether the addition of LUS to current pneumonia pathways in Mozambique and Pakistan improves identification of child pneumonia, and whether LUS is feasible, usable and acceptable among healthcare providers and caregivers

in these settings. Based on the results from this pilot study, we will be able to determine the need for a larger, more robust evaluation.

3 STUDY HYPOTHESIS, OBJECTIVES AND ENDPOINTS

3.1 Study Hypothesis

- The addition of LUS to the current WHO IMCI guidelines will improve identification of pneumonia in children 2 through 23 months of age.
- LUS is feasible, usable and acceptable among healthcare providers and caregivers of children with pneumonia
- The addition of LUS to the current WHO IMCI guidelines may improve management of pneumonia by identifying children with higher risk of progression.

3.2 Study Objectives

Primary Objective:

- To provide scientific evidence assessing whether the addition of LUS to the current pneumonia pathways in Mozambique and Pakistan improves identification of pneumonia in children 2 through 23 months of age presenting to district hospitals
- To determine whether LUS is feasible, usable and acceptable among healthcare providers and caregivers of children with respiratory symptoms for diagnosing and managing pneumonia.

Secondary Objectives:

- To assess whether the specificity of the WHO IMCI criteria increases when LUS is added to the current diagnostic pathway
- To assess whether LUS may be able to help characterize and prioritize which children require hospitalization or are at higher risk for progression of the pneumonia or their acute process
- To investigate whether there are different characteristic LUS imaging patterns between viral, bacterial and mixed pneumonia

3.3 Study Endpoints

Primary Endpoints:

- Proportion of children with pneumonia suggested by LUS and/or CXR

Secondary Endpoints:

- Proportion of children with pneumonia suggested by CXR but not LUS

- Proportion of children with pneumonia suggested by LUS but not CXR
- Proportion of children with no pneumonia identified
- Etiological diagnosis for each recruited child with samples collected
- Proportion of children with a positive viral PCR
- Difference in characteristic LUS imaging patterns between children with viral versus bacterial versus mixed pneumonia
- Clinical and/or diagnostic biomarker outcomes
- Patient status after 6 days of follow-up
- Patient status after 14 days of follow-up
- Patient status after 30 days of follow-up
- Feasibility, usability and acceptability of LUS among HCPs
- Acceptability of LUS among caregivers

4 METHODOLOGY

4.1 Study Design

This project involves a facility-based observational study at one district hospital each in Manhiça, Mozambique and Karachi, Pakistan, assessing the utility of point-of-care LUS to diagnose childhood pneumonia in LRS. Cases presenting with cough <14 days and/or difficulty breathing <u>and</u> chest indrawing, and controls presenting with cough <14 days and/or difficulty breathing with no chest indrawing, fast breathing or fever will be enrolled at a 5:1 ratio.

All enrolled children will receive local standard of care including WHO IMCI assessment and management as well as CXR and LUS at enrollment. A respiratory specimen and blood sample for pneumonia etiology characterization may also be collected from all children at enrollment. Panels of experts will be assembled to interpret the CXR and LUS results to ensure consistency and accuracy of interpretation. Enrolled children will be followed through hospital outcome, as well as 14 days (in person) and 30 days (phone call) post-enrollment, with repeat LUS evaluations on days 2, 6, and 14. The day 30 phone call is intended to determine the child's health status. Should the phone call detect any referred ongoing clinical problem, the child would be visited in person by a study team member or study clinician. Management of children will be expedited according to the clinician's judgment of each child. Children will receive the first-line treatment for pneumonia at each site, according to national guidelines. Under no circumstances will the participation in the study interfere with or unnecessarily delay the management of sick children.

We will also conduct a mixed methods evaluation to assess the feasibility, usability, and acceptability of LUS for diagnosing childhood pneumonia in a LRS, including in-depth interviews (IDIs) and/or structured questionnaires. All PLUS study HCPs at both sites may be involved in this portion of the study. Healthcare administrators may also be invited to participate. We anticipate that total participation will not exceed ten HCPs and administrators per site. IDIs and direct

observation will take place at each site to assess the acceptability of LUS. We anticipate that up to 24 caregivers will participate in this portion of the study.

4.2 Study Site

For this project, Save the Children Federation Inc., United States is the responsible lead partner and principal investigator (PI), coordinating closely with co-investigators from Barcelona Institute for Global Health (ISGlobal) in collaboration with Centro de Investigação em Saúde de Manhiça (CISM), Aga Khan University, Pakistan (AKU-P), and San Luigi Gonzaga University (SLGU).

Research will take place at the Centro de Investigação em Saúde da Manhiça (CISM) in Manhiça, Mozambique and the Sindh Government Children's Hospital (SGCH) in Karachi, Pakistan. Details about each study site are as follows:

CISM was created in 1996 with the objective of conducting biomedical research in those diseases that affect the most poor and vulnerable. CISM works closely with the Manhiça District Hospital, the referral health facility for the entire Manhiça District in Mozambique. This public hospital includes 32 beds in the pediatric ward, 8 beds in a basic intensive care facility, and 6 beds in a day hospital where children can be temporarily admitted and observed prior to a final admission decision. The hospital has a fully digital (film-free) CXR machine, and a clinical trials unit. Further description of this study site can be found in Appendix V: Specific Information Regarding the Manhiça Site. Note that this appendix is specific to the Mozambique site only; details in this appendix do not apply to the Pakistan site.

SGCH was created in 2003 as a district hospital providing health care services free of cost to children from LRS. SGCH is located in District Central and serves the largest district of Karachi, Pakistan. Recently, SGCH has become a public-private partnership with the Poverty Eradication Initiative (PEI), a private non-profit organization that assists the Government of Sindh in managing the hospital. This public hospital has 50 functional beds, an operation theatre, an intensive care unit (ventilators currently not functioning) and a neonatal unit. Other established services include surgery and ENT. The hospital has a fully digital (film-free) CXR machine and a laboratory. There is a daily influx of about 1500 children in the emergency and outpatient clinics. SGCH has been a sentinel site for pneumonia and meningitis surveillance to study the effectiveness of first Hib and then pneumococcal vaccines. Based on previous studies, it is estimated that 15-20 cases of chest indrawing pneumonia can be enrolled every week.

4.3 Study Population

4.3.1 Study Population Overview

Manhiça, Mozambique: Manhiça is located in southern Mozambique, about 90 km from

Mozambique's capitol, Maputo. The country is ranked 180th of 188 countries in the United Nations Development Programme's Human Development Index (HDI) and has a gross national income (GNI) per capita of $580 USD/year (2015).[52] Life expectancy at birth is estimated to be 55 years.[53] The WHO has estimated the 2013 under-five mortality rate to be 87.2/1000 live births; 19% of post-neonatal deaths (aged 1-59 months) were estimated to be caused by pneumonia.[54]

While Mozambique's adult HIV prevalence is estimated to be 10.5%, the burden is significantly higher in Manhiça, with a 2012 estimate indicating 39.9% seropositivity among adults.[55] In 2015, there were an estimated 110,000 children aged 0-14 living with HIV. [56] Malaria is endemic in Mozambique, and its burden is greatest in rural areas: according to the 2011 Demographic Health Survey, the malaria prevalence in rural areas was 46%, nearly three times the 16% prevalence recorded in urban areas.[57]

Karachi, Pakistan: Karachi is the largest and most populous city in Pakistan. Pakistan is ranked 147th in the HDI, and has a GNI per capita of $1440 USD/year.[52] Life expectancy at birth is estimated to be 66 years.[58] The 2013 under-five mortality rate was estimated to be 85.5/1000 live births; pneumonia was the number one cause of post-neonatal deaths, at 29%.[59] The HIV burden in Pakistan is low, with an adult prevalence of <0.1%.[60] While malaria is endemic in Pakistan, its burden varies geographically, with low transmission in the Karachi area (defined as prevalence of 0-1 case per 1,000 people.[61,62]

We expect enrolled children at each site to be representative of the ethnic demographics in the both areas. We anticipate enrolling equal numbers of female and male children.

4.3.2 Participant Eligibility

Cases will be children 2 through 23 months of age who present to a study hospital with history of cough or difficulty breathing and chest indrawing. Controls will be children 2 through 23 months of age who present with cough or difficulty breathing, but without chest indrawing, fast breathing or fever. Cases and controls may be inpatient or outpatient at the discretion of the study site.

Volunteer families will be recruited and screened, and those whose children are determined to be eligible based on the inclusion/exclusion criteria will be enrolled in the study and followed for 14 days in person and 30 days via phone, or, if not available, by a home visit. Final eligibility determination will depend on the results of the medical history, clinical examination, appropriate understanding of the study and completion of the consent process.

Inclusion Criteria, Cases

1. Male or female, 2 through 23 months of age
2. Cough <14 days or difficulty breathing

3. Visible indrawing of the chest wall, with or without fast breathing
4. Ability and willingness of child's caregiver to provide informed consent and to be available for follow-up for the planned duration of the study, including accepting a home visit if he/she fails to return to the study facility for a scheduled follow-up visit

Exclusion Criteria, Cases

1. Presence of WHO IMCI danger signs including lethargy or unconsciousness, convulsions, vomiting everything, or inability to drink or breastfeed
2. Presence of respiratory danger signs (e.g., head nodding, nasal flaring or grunting)
3. Known or possible tuberculosis (TB) (history of a cough ≥14 days)
4. Oxygen saturation <90% on room air
5. Stridor when calm
6. Chest indrawing observed at screening resolves after bronchodilator challenge (among those with wheeze at screening)
7. Living outside the study catchment area

Inclusion Criteria, Controls

1. Male or female, 2 through 23 months of age
2. Cough <14 days or difficulty breathing
3. Ability and willingness of child's caregiver to provide informed consent and to be available for follow-up for the planned duration of the study, including accepting a home visit if he/she fails to return to the study facility for a scheduled follow-up visit

Exclusion Criteria, Controls

1. Presence of fast breathing, defined as a respiratory rate ≥50 breaths per minute in children aged 2 through 11 months of age or ≥40 breaths per minute in children aged 12 through 23 months
2. Visible indrawing of the chest wall
3. Measured fever ≥38° C
4. Presence of WHO IMCI danger signs including lethargy or unconsciousness, convulsions, vomiting everything, or inability to drink or breastfeed
5. Presence of respiratory danger signs (e.g., head nodding, nasal flaring or grunting)
6. Known or possible TB (history of a cough ≥14 days)
7. Oxygen saturation <95% on room air
8. Stridor when calm
9. Any medical or psychosocial condition or circumstance that, in the opinion of the investigators, would interfere with the conduct of the study or for which study participation might jeopardize the child's health
10. Living outside the study catchment area

For the feasibility, usability and acceptability assessment, PLUS study HCPs will be enrolled if they are 18 years or older, involved in or aware of the PLUS study, and have provided written informed consent. Caregivers will be enrolled if they are 18 years or older, have a child enrolled in the study, and are willing to participate in a 30-minute IDI as well as direct observation while LUS is performed on their child.

*Note: Recruitment of cases in Mozambique will include hospitalization, according to the local standard of care, while recruitment of controls may be done at the outpatient department, with no need for hospitalization.

4.3.3 Sample Size

A total of 270 children will be enrolled. This will comprise 100 cases and 20 controls at the Mozambique site and 130 cases and 20 controls at the Pakistan site. This is a pilot study seeking to demonstrate feasibility of investigating LUS for pneumonia diagnosis and prognosis in a sub-district facility setting. Its underlying purpose is to generate evidence to inform future full-scale studies. Thus, the sample size may not provide adequate power to answer all of the research questions laid out in this protocol.

Academic guidance on the subject of sample size in pilot studies varies.[63-66] Some articles indicate that an appropriate sample size for a pilot study should be 10% that of a fully-powered study; a 2012 audit of sample sizes in pilot and feasibility studies found a median sample size of 36 participants per arm for trials with a dichotomous outcome.[63-65] The sample size for this study (200 cases and 40 controls) was selected to maximize the amount of information collected within the confines of the available resources. With the proposed 200 case participants, if a low estimate of 30-40% of enrolled children with chest indrawing were found to have pneumonia by CXR, we would still have a sample of 60 to 80 case participants with pneumonia. We believe that this is sufficient to generate evidence and inform future studies regarding the use of LUS as a tool for pneumonia diagnosis and prognosis.

For the feasibility, usability, and acceptability assessment, all PLUS study HCPs will be asked to participate in the data collection procedures; healthcare administrators may also be invited to participate. We anticipate that the sample size will not exceed 10 HCPs per site. Caregivers who consent to participate in the acceptability assessment will participate in an IDI as well as direct observation while LUS is performed on their child. Up to 24 caregivers will participate at each site.

4.4 Study Period

Following enrollment, each child will be followed for 14 days in person and 30 days via phone. Total duration of a child's participation in the study is 30 days. Projected duration of enrollment is anticipated to be about 12 months for this study.

5 STUDY PROCEDURES

Refer to Appendix I for **Study Flow Diagram** and Appendix II for **Study Procedures and Visits Table.**

5.1 Recruitment

Recruitment for this study will be performed by hospital staff during routine intake and screening procedures. In Pakistan, recruitment will take place in the emergency department and outpatient clinics at SGCH. In Manhiça, recruitment will take place at Manhiça District Hospital and Health Centre. Children between 2 through 23 months of age presenting with cough or difficulty breathing will be assessed by hospital staff for potential referral to the study. For any children whose cough is less than 14 days duration or reports difficulty breathing, the clinician will provide a brief introduction to the study. If the caregiver is interested in learning more about the study and in potentially having the child assessed for eligibility, he/she will be referred to PLUS study staff.

All hospital staff involved in PLUS study recruitment procedures will be trained in relevant study-specific procedures and certified in good clinical practice (GCP). Each recruitment and referral interaction will be documented for study records.

5.2 Screening

Screening procedures are conducted by PLUS study staff to determine eligibility for enrollment in the study. To avoid potential selection bias, each day children will be screened for enrollment in a sequential manner, as much as possible. All inclusion/exclusion criteria must be assessed on presentation. The following procedures will be performed by PLUS study staff for screening:

- Provide information on the study
- Assess all eligibility criteria, including chest indrawing, assessment of stridor when calm and assessment of general and respiratory danger signs
- Collect medical history
- Perform pulse oximetry to assess hypoxia
- Measure temperature
- Count respiratory rate
- Collect demographic and address information

For those children who are not eligible, study staff will inform the caregiver(s) that their child will not be able to participate in the study and will receive local standard care instead. All screening procedures will be documented in the appropriate study forms, including logs and case report forms. Clinical assessments and findings will also be documented in the child's medical record, as appropriate. No identifying information will be retained for children who do not enroll in the study.

5.3 Informed Consent Process

For the purposes of this protocol, "caregiver" refers to the child's parent or guardian. Informed consent will be obtained from each child's caregiver to ensure that the caregiver is informed of and fully understands what will and may happen to their child while participating in a research study. PLUS study staff will administer a comprehension checklist to potential participants' caregivers prior to obtaining written informed consent to ensure that caregivers fully comprehend the nature of the study. The informed consent process continues throughout the study. Key study concepts will be reviewed periodically with the caregivers. Additionally, if any new information is learned that may affect the caregiver's decision to stay in the study, this information will be shared with the caregivers in writing. All consent materials will be approved by the appropriate Institutional Review Board (IRB) and Independent Ethics Committee (IEC) prior to use.

Written informed consent will be collected from all HCPs and administrators participating in the qualitative portion of the study. HCPs and administrators may decline to participate without any negative effects on their employment. Caregivers may decline to participate in the qualitative portion of the study and still have their child participate in the parent study.

Refer to detailed description of informed consent procedures and ethics committee approval in Section 10 (Ethical Considerations and Consent).

5.4 Enrollment Visit

After screening is complete, PLUS study staff will perform the enrollment visit procedures for those children who are still eligible. The following procedures will be performed at enrollment:

Cases:

- Obtain written informed consent for enrollment
- Assign participant identification (ID) study number
- Collect additional medical history and socio-demographic information not already collected during screening
- Collect information on environmental exposures
- Collect vaccination history
- Collect information on concomitant medications and antibiotic use
- Collect information on current illness
- Collect information on pneumonia hospitalization
- Perform a physical exam including vital signs and an assessment of any baseline characteristics not already recorded in the medical record or assessed during screening
- Collect information on severe acute malnutrition (SAM), including weight for length and mid upper arm circumference (MUAC)
- Administer LUS (within 8 hours after the study physical exam)

- Administer CXR (within 8 hours after the study physical exam)
- Collect respiratory specimen, as applicable
- Collect blood sample for hemoglobin (Mozambique, Pakistan), malaria (Mozambique only) and HIV screening (Mozambique only), as well as for bacterial invasive disease screening, as applicable (see Appendix III for more information on HIV screening at the Mozambique site)
- Collect locator information to be able to contact caregiver and conduct a home visit, if necessary

Controls:

- Obtain written informed consent for enrollment
- Assign participant ID number
- Collect additional medical history and socio-demographic information not already collected during screening
- Collect information on environmental exposures
- Collect vaccination history
- Collect information on concomitant medications and antibiotic use
- Collect information on current illness
- Collect information on pneumonia hospitalization
- Perform a physical exam including vital signs and an assessment of any baseline characteristics not already recorded in the medical record or assessed during screening
- Collect information on SAM, including weight for length and MUACAdminister LUS (within +8 hours of the study physical exam)
- Administer CXR (within +8 hours of the study physical exam)
- Collect respiratory specimen, as applicable
- Perform finger stick or collect blood sample for hemoglobin (Mozambique, Pakistan), malaria (Mozambique only) and HIV screening (Mozambique only) (see Appendix III for more information on HIV screening at the Mozambique site)
- Collect locator information to be able to contact caregiver and conduct a home visit, if necessary

All enrollment procedures will be documented in the appropriate study forms. Clinical assessments and findings will also be documented in the child's medical record, as appropriate.

5.5 Follow-Up Visits

Target dates for follow-up visits are calculated from Day 1, the date of enrollment. All visits must occur on the calendar day on which they are initially scheduled or within 24 hours afterwards, with the exception of the Day 14 visit and the Day 30 phone call, which can occur 72 hours before or after Day 14 and still be considered completed within the visit window.

If the child is not still in the hospital during the follow-up windows, PLUS study staff will attempt to contact the caregiver by phone prior to scheduled study visits to remind them to return to the clinic at the appropriate time. Pick up and drop off may be provided at site discretion.

Follow-up visit procedures at scheduled visits include the following:

- Review/update locator information
- Update medical history
- Update concomitant medications and antibiotic use
- Update current illness
- Update pneumonia hospitalization
- Targeted physical exam (except Day 30 visit)
- Assess for general and respiratory danger signs (except Day 30 visit)
- Repeat LUS (except Day 30 visit)
- Refer child to clinical care, as needed

All follow-up visit procedures will be documented in the appropriate study forms. Clinical assessments and findings will also be documented in the child's medical record, as appropriate.

5.6 Unscheduled Visits

If a child presents to a study hospital during the period of his/her participation, an unscheduled visit may be performed. Unscheduled visits may include the following:

- Review/update locator information
- Update medical history
- Update concomitant medications and antibiotic use
- Update current illness
- Update pneumonia hospitalization
- Targeted physical exam
- Assess for general and respiratory danger signs
- Repeat LUS
- Refer child to further clinical care, as needed

All unscheduled visit procedures will be documented in the appropriate study forms. Clinical assessments and findings will also be documented in the child's medical record, as appropriate. Unscheduled visits will not prevent or delay the child's care.

5.7 Missed Visits

In case of a no-show for a scheduled study visit, PLUS study personnel will call the caregiver and visit the child's home either that same day or the following day to conduct the study visit. Maximum efforts will be made to ensure complete follow-up in the study. For children who do

not complete a scheduled visit within the visit window, that visit will be documented as "missed" but PLUS study staff will still attempt to complete the appropriate assessments from that visit, if possible (e.g. Day 6 visit performed and documented on Day 9). Children who miss a visit are permitted to continue with any subsequent study assessment that can still be scheduled in the time interval specified by the protocol.

Based on our current experience, we expect that fewer than 10% of the children will be lost to follow-up. We think it is unlikely that attrition rates will differ between cases and controls. The primary outcome of this study may be assessed even for children who are lost to follow-up.

5.8 Withdrawal and Early Termination

Children and their caregivers may voluntarily withdraw from the study for any reason at any time. The site investigators may also withdraw children from the study in order to protect their safety if, in the investigators' opinion, continuing participation would jeopardize the child's health. Any participant withdrawal or early termination will be documented in the appropriate study forms.

5.9 Study Termination Visit

The Day 30 visit (phone) will serve as the study termination visit. Procedures for this visit include the following:

- Update medical history
- Update concomitant medications and antibiotic use
- End of study questions
- Refer child to clinical care, as needed
- Document contact in child's study records

5.10 Participant Reimbursement

Appropriate reimbursement will be determined by local investigators at each site. Caregivers may also receive reimbursement for participation in IDIs, at the discretion of local investigators. Reimbursement will be payable at the end of each visit. The study consent form will list the minimum amount to be paid in the local currency.

At the Karachi site, travel reimbursement will be provided to caregivers to compensate them for the cost of transportation for study visits and IDI participation, up to PKR 1000 (10 USD). Alternatively, transport to and from the study site will be provided by the study.

Study participants' caregivers will not be responsible for paying for PLUS study-related examinations.

6 RADIOLOGY AND SPECIMENS

6.1 LUS Collection and Interpretation

During the LUS examination, the child's lungs will be examined by longitudinal and oblique scans of the anterior, lateral and posterior chest. Six areas will be examined on each enrolled child, comprising the anterior, lateral and posterior areas of the lungs, further divided into the upper and lower halves. The number of scans required will depend on the body size of the child; for smaller children, one anterior, one lateral and one posterior scan may be sufficient to cover the child's whole lung surface per side. The child will be examined in his/her most comfortable position (e.g., the caregiver's arms).

To ensure quality and consistency of LUS interpretation, the LUS Standard Operating Procedures (SOP) will provide an *a priori* description of features typical to pneumonia diagnosis, as well as guidance toward identifying secondary outcomes (including severe pneumonia and viral versus bacterial versus mixed pneumonia). The LUS interpretation will be targeted to the detection of typical subpleural lung consolidations with tissue-like or anechoic patterns and blurred, irregular margins. LUS will be considered positive when at least one consolidation showing features described in the LUS SOP is present on imaging. At least two independent LUS-trained PLUS study team members will interpret each LUS. If discordant, a designated LUS expert will act as a tiebreaker.

Additional details regarding LUS collection and interpretation are described in brief below:
Following completion of the LUS examination, the PLUS study team member will complete a standardized form reporting all chest areas examined as well as the patterns detected, including interstitial syndrome, consolidation and effusion. Reporting of adjunctive signs will include the following:

a) Respiratory sliding
b) Air bronchograms
c) Fluid bronchograms
d) If consolidation is present,
    a. Shape (regular versus irregular)
    b. Quality of margins (sharp versus blurred)
    c. Echotexture (anechoic versus tissue-like)
e) If effusion is present, complexity (anechoic versus echoic)

This study will also investigate LUS features hypothesized to be correlated with severity of pneumonia. To this aim, each child with consolidation on LUS imaging will have the following information collected:

- Location of the consolidation
- Dimension of the consolidation
- Multifocality/bilaterality
- Presence of pleural effusion
- Dimension of pleural effusion and consolidation/effusion ratio
- Echogenicity of effusion/complexity of effusion.

6.2 CXR Collection and Examination

For the purposes of this protocol, CXR will be considered the reference standard for pneumonia diagnosis. CXR images will be collected based on the current standard practice at each study site.

The CXR interpretation panel will investigate radiographic indicators of primary end-point pneumonia, in a process modeled after the WHO CXR process.[67] Interpretation will focus on the presence of consolidation, infiltrates, and/or effusion.

At least two independent CXR-trained study PLUS team members will interpret each CXR. If discordant, a designated CXR expert will act as tiebreaker. CXR collection and interpretation is further described and detailed in the CXR SOP.

6.3 Specimen Collection

Respiratory specimens may be collected from children enrolled as cases and controls, at the discretion of study investigators, after consent is given. The Mozambique site may collect one nasopharyngeal aspirate (NPA) per child for viral testing and/or one pharyngeal swab per child for bacterial culture. The Pakistan site may collect one nasopharyngeal (NP) swab per child for viral testing and bacterial culture. All respiratory samples will be collected based on local standard practice at each study site.

A blood sample may also be collected from children enrolled as cases and controls. For cases, the blood sample may be used for blood culture and for biomarker identification. For children enrolled as controls, a finger stick may suffice, at the discretion of local investigators. Whenever possible, the research blood draw will be combined with a planned clinical blood draw to avoid multiple needle sticks. At SGCH, blood will be collected either by finger stick or through venipuncture if clinically indicated. This blood will be used to measure hemoglobin. In any case, the maximum volume of blood obtained during the enrollment visit will be 3.5 mL.

For additional information and instructions, see the Sample Collection, Transport, Processing and Testing SOP. For additional information regarding sample collection at the Mozambique site, see Appendix III. This appendix also specifies the rationale for sample collection, and summarizes in a table all samples and volumes needed at the Mozambique site.

6.4 Specimen Transport

All specimens must be labeled with the participant ID prior to transport. Specimens will be transported based on local guidelines and standard practice at each site. Samples will be maintained at the proper temperature at all times. For all specimens, cold chain information will be documented and will include the time the sample was collected, the time the sample was put on ice and/or in the refrigerator, and the time the sample arrived at the laboratory (as applicable).

For additional information and instructions, see the Sample Collection, Transport, Processing and Testing SOP.

6.5 Specimen Processing and Testing

All specimens will be processed based on standard procedures at each study site.

NPA samples collected at the Mozambique site and NP swabs collected at the Pakistan site will be tested for respiratory viruses using a multiplex RT-PCR panel. Pharyngeal swabs collected in Mozambique will undergo bacterial culture for pathogens including *Streptococcus pneumoniae* and *Haemophilus influenzae.*

Blood collected from cases and controls will undergo hemoglobin testing at both study sites, as well as HIV and malaria testing at the Mozambique site only. Blood collected from cases may also undergo bacterial culture and/or biomarker investigation, as appropriate.

For a detailed description of sample processing and testing, as well as counseling, treatment and care for HIV-positive individuals at the Mozambique site, see Appendix III.

7 DATA COLLECTION

Clinical research data will be maintained through a combination of secure electronic data management system and physical files with restricted access. Data related to study endpoints will be extracted from the electronic databases for statistical analysis. The database linking children's identifiable information to their participant ID, and any other documentation (paper-based or electronic) that has both personal identifiers and the participant ID will have restricted access and will be stored in a secure manner separately from other study data. This database will be retained for at least five years after the last participating child exits the study.

7.1 Case Report Forms

All study data will be collected by PLUS study staff using designated source documents or case report forms (CRFs). Study data will be entered directly into the CRFs during a study visit. Data from the CRFs will be entered into the electronic database as promptly as is feasible. PLUS study

staff will maintain source documents for each child at the study site. Source documentation will be available for review to ensure that the collected data are consistent with the CRFs. CRFs, source documents and other supporting documents (both electronic and paper-based) will be kept in a secure location and remain separate from participant identification information (name, address, etc.) to ensure confidentiality. GCP will be followed to ensure accurate, reliable and consistent data collection.

7.2 Source Documents

Source documents include but are not limited to:

- Signed informed consent forms (ICFs)
- Documentation of the comprehension checklist
- Visit documentation that includes dates of study visits
- Receipts for travel reimbursement
- Clinical notes

Site investigators will maintain, and store in a secure manner, all source documents throughout the study. These documents will be retained for at least five years after the last child exits the study.

7.3 Data Management

Local data management will take place at each study site, with support from SC-US. Data management activities include data entry and validation, data cleaning, database quality control, disaster recovery plans, preparation and submission of compliance reports to the funding agency, and preparation of the final study database.

7.4 Data Access

The participating sites will maintain appropriate medical and research records for this study, in compliance with GCP, regulatory, sponsoring organization and institutional requirements for the protection of confidentiality of children. The site will permit authorized representatives of the sponsor and regulatory agencies to examine (and when required by applicable law, to copy) clinical records for the purposes of quality assurance reviews, audits and evaluation of the study safety and progress. User rights will be provided to PLUS study staff, PIs, and co-investigators at the level appropriate for each individual's job description.

7.5 Data Storage

The site investigators and designees will maintain, and store securely, complete, accurate and current study records throughout the study. PLUS study staff will retain all study records on site for at least five years after study closure. Study records will not be destroyed prior to receiving approval for record destruction from the sponsor. Applicable records include source documents, ICFs, and notations of all contacts with the child.

8 TRAINING REQUIREMENTS, SAFETY ASSESSMENTS AND REPORTING

8.1 Training Requirements

All PLUS study staff will be trained in the Protection of Human Subjects and GCP prior to any interactions with PLUS study participants. Prior to study initiation, all PLUS study staff will receive training to review all study procedures, including the study protocol, SOPs, data collection tools, informed consent process and reporting. PLUS study staff involved in case-finding will be trained to perform the WHO IMCI assessment and to identify and assess inclusion/exclusion criteria (including identifying chest indrawing, counting respiratory rate, performing pulse oximetry, assessing for danger signs, etc.). Trainings will be conducted by a representative of the study consortium or other qualified clinician, as appropriate for the training material.

PLUS LUS and CXR technicians will receive training regarding technique; PLUS LUS and CXR interpreters will receive training regarding patterns to be annotated for the evaluation of pneumonia and severe disease. Regular quality control (QC) of LUS and CXR will take place, along with testing and refresher trainings. See the detailed LUS SOP and CXR SOP for additional information.

Both PLUS study staff and general hospital staff at both sites will be sensitized to this study. PLUS study staff will receive at least one day of training on the identification of eligibility criteria and study-specific procedures and documentation prior to the study start. Refresher trainings will be held periodically, at least once every year. Due to the technical nature of this study, local staff and facilities will benefit from the capacity building involved in LUS and CXR training. As these technologies become increasingly available in LRS, this technical experience will provide improved capacity for diagnosis and case management as well as for career development among local staff.

8.2 Monitoring

The study site investigators will be responsible for close safety monitoring of all children participating in the study, and for alerting the protocol team if unexpected concerns arise. All children will undergo a targeted physical exam at screening and enrollment to ensure that children are medically stable and do not demonstrate any exclusion criteria. Each participating child will be evaluated by a study clinician at each in-person study visit. If a child misses an in-person study visit, home visits will be conducted by trained PLUS study staff to ensure clinical evaluation.

Study investigators will hold regular conference calls to monitor progress and ensure homogeneity in protocol execution.

8.3 Study Discontinuation

The study may be discontinued at any time by the protocol team, funding agency, regulatory authorities, or institutional review board/ethics committee.

9 STATISTICAL DESIGN AND ANALYSIS

9.1 Overview and General Design

In brief, we plan to conduct a facility-based, prospective, observational study of the impact of LUS on the management of pneumonia among children 2 through 23 months presenting for care at Manhiça District Hospital and Health Centre (Mozambique) or SGCH (Pakistan). This study will enroll a total of 270 children. All children will present with cough <14 days and/or difficulty breathing; cases will present with chest indrawing and controls will present without chest indrawing, fast breathing or fever. At the Mozambique site, 100 cases and 20 controls will be enrolled. At the Pakistan site, 130 cases and 20 controls will be enrolled. Each child will receive standard of care per local guidelines and/or ward protocol, will undergo CXR and LUS at enrollment, and may have a respiratory specimen and blood sample collected at enrollment. Children will be followed for a total of 14 days in person and 30 days via phone, and will have repeat LUS assessments at days 2, 6 and 14.

We will also conduct a mixed methods evaluation to assess the feasibility, usability, and acceptability of LUS for diagnosing childhood pneumonia in a LRS, including IDIs and/or structured questionnaires and direct observation. All PLUS study HCPs at both sites may be involved in this portion of the study; healthcare administrators may be invited to participate as well. Up to 24 caregivers of children assessed for pneumonia with LUS will participate in the caregiver assessment.

9.2 Hypothesis

We hypothesize (stated under the alternative):

- For the diagnosis of CXR-confirmed pneumonia (radiological endpoint pneumonia), the specificity of the WHO IMCI clinical assessment algorithm plus LUS will be greater than the specificity of the WHO IMCI clinical assessment algorithm alone.

We will also conduct exploratory investigations regarding whether LUS may be able to help characterize and prioritize which children require hospitalization or are at higher risk of progression of their pneumonia or acute process, whether LUS can identify characteristic imaging differences in viral versus bacterial versus mixed pneumonia, and whether LUS is feasible, usable and acceptable among HCPs and caregivers for diagnosing pediatric pneumonia in a LRS.

9.3 Analytical Methodology

McNemar's test of paired data will be used to compare discordance in results between LUS and the WHO IMCI clinical assessment algorithm. This will be performed as a two-sided test with alpha = 0.05. To assess sensitivity and specificity, Receiver Operating Characteristic (ROC) curves will be calculated, and the area under the curve (AUC) will be compared between tests. Likelihood ratios will also be reported. Interrater agreement for CXR and LUS will be determined using kappa statistics.

We anticipate that some children may not return for their scheduled follow-up visits, and we have estimated the loss to follow-up to be 10% in this study. The primary hypothesis for this study can be assessed even for children who do not return for follow-up.

Feasibility, usability, and acceptability will be assessed through qualitative data analysis of IDIs with HCPs and caregivers, as well as direct observation with caregivers. The qualitative data will be in narrative format and the results will be descriptive. The transcripts will be coded and analyzed using a codebook and themes identified a priori. This codebook will address several themes, which may include opportunities and barriers for introduction of LUS, feasibility of implementing LUS, and perceived value. Qualitative data analysis software will be used to organize, code, and analyze the qualitative data. The data will be analyzed in an iterative process. The research team will start by identifying an initial set of codes and themes based on the categories from the IDI and guides. During the coding process, attention will be paid to identifying emergent issues and themes that will be added to the codebook and included in the analysis. Transcripts of the IDIs will be coded and discrepancies will be discussed and resolved for the final analysis and theme identification.

9.4 Result Presentation

Results of this research will be primarily presented through at least one published manuscript with detailed description of the background, methods, results, discussion and conclusions. The specific format and details of this manuscript will be in accordance with the requirements of the publishing journal.

9.5 Dissemination of Results

The results of this study will be published collaboratively by investigators at SC-US, SLGU, ISGlobal/CISM and AKU-P in peer-reviewed journals. Study findings will be presented to staff at each study site. Study results will be presented at least one international conference to disseminate the findings of the study.

10 ETHICAL CONSIDERATIONS AND CONSENT

10.1 Principles for Clinical Research

This clinical study will be conducted in compliance with the protocol, GCP, and all applicable

regulatory requirements and IRB/IEC reviews. All study activities will follow the ethical principles of the Declaration of Helsinki.[68] All PLUS study staff will be trained and certified in the protection of human subjects.

10.2 Institutional Review Boards (IRBs) and Independent Ethics Committees (IECs)

The IRB of record for this study is the Western Institutional Review Board (WIRB). A copy of the protocol, proposed ICFs, other written participant information, and any proposed advertising materials will be submitted to WIRB for written approval. The investigators must submit and, where necessary, obtain approval from the IRB/IEC at their local institution for the initiation of the study and all subsequent protocol amendments and changes to the ICF. SC-US is responsible for assuring that this protocol, ICFs and any other study-related documents are approved by WIRB prior to implementation of the protocol. Any subsequent amendments to the protocol or other study-related documents must be approved by WIRB prior to implementation. The study will be conducted in full compliance with the protocol. Any deviations from or violations of the protocol will be documented and submitted to the appropriate IRB/IEC by investigators as required. The protocol will not be amended without prior written approval by the PI.

10.3 Informed Consent Documentation

In obtaining and documenting informed consent, the site investigators and their designees will comply with applicable local and domestic regulatory requirements and will adhere to GCP. English, Portuguese (Mozambique), Changana (Mozambique), Urdu (Pakistan) and Sindhi (Pakistan) versions of the ICF will be reviewed and approved by the appropriate IRB/IEC before use with participants' caregivers. The ICF will include the purpose of the study, a description of the procedures to be followed and the risks and benefits of participation. The informed consent process will give individuals all of the relevant information necessary to decide whether to participate, or to continue participation, in this study. Potential research participants' caregivers will be permitted to ask questions and to exchange information freely with the study team. If the caregiver providing consent is illiterate, an independent witness will be present to verify to the caregiver that all the information read aloud is contained in the ICF. In this instance, the caregivers will thumbprint the ICF, which will be countersigned by the impartial witness.

Before a child begins participation in the study, it is the site investigators' responsibility to ensure that informed consent is obtained from their caregiver after adequate explanation of the aims, methods, and potential risks and benefits of the study. The PLUS study staff obtaining consent will also sign and date the ICF. A signed and dated copy of the consent form will be given to the participant's caregiver and this will be documented in the child's study record.

10.4 Risks and Benefits

10.4.1 Risks to Participants

Coercion

Caregivers may feel coerced to enroll in the study in order to receive care for their child within a research setting, which may be perceived as of a higher quality than the standard of care.

Specimen Collection
The study may involve the collection of a respiratory specimen and a blood sample at enrollment. Collection of a respiratory specimen may cause discomfort, and may cause the child to cough or sneeze. In rare cases, collection of a respiratory specimen may cause the nose to bleed. Collection of blood by venipuncture or finger stick may cause minimal discomfort in the child.

CXR assessment
The collection of CXR may expose children to a low level of ionizing radiation, and may cause the child minor, temporary or distress while being held still for the examination.

LUS
The collection of LUS may cause the child minor, temporary distress while being held still for the examination.

Medical Management
Participation in the study has the potential to compromise care for hospitalized children, if study procedures are prioritized above urgent clinical care for acute infections. Study staff will guarantee that this will not be the case, and children may be excluded if study staff believes that including them in the study could jeopardize their prompt medical attention.

10.4.2 Protection against Risks

Coercion
During the informed consent process, PLUS study staff will emphasize that the study is optional, and that the child will receive medical care whether enrolled in the study or not.

Specimen Collection
In order to minimize the risks associated with blood and respiratory specimen collection, all PLUS study staff who will be collecting specimens from children in the study will be trained in the appropriate procedures and supervised accordingly. Whenever possible, research blood draws will be combined with clinical blood draws to minimize the amount of needle sticks experienced by the child.

CXR assessment
All PLUS study staff implementing CXR will be trained in appropriate procedures and supervised accordingly. Standard precautions will be in place to protect children from radiation. To

minimize the dose of ionizing radiation, only one CXR will be obtained during the study period; there will be no repeat CXR assessments unless clinically indicated.

LUS assessment

All PLUS study staff implementing LUS will be trained in appropriate procedures and supervised accordingly. Standard precautions will be in place. The child will be examined in his/her most comfortable position (e.g. in caregiver's arms).

Medical Management

In order to minimize the possibility that participation in this study will interfere with the medical management of children who are hospitalized with pneumonia, PLUS study staff will be trained in integrating research procedures with clinical care. Urgent clinical care for acute medical issues will always be prioritized above research procedures.

10.4.3 Benefits to Participants

Direct benefits to children in this study include increased clinical supervision and care during the study period. Frequent follow-up visits are not included as standard of care, so participating children will benefit from monitoring for two weeks from the illness episode, and 30 days via phone.

If this study demonstrates superior specificity of LUS, the results have the potential to inform and support national and international guidelines for duration of treatment for childhood pneumonia.

10.5 Participant Confidentiality

The site investigators must ensure that the child's confidentiality is maintained. Personal identifiers will not be included in any study reports. All study records will be kept confidential in keeping with IRB/IEC regulations as well as national and local laws. All study procedures will be conducted in such a manner as to protect participant privacy and confidentiality to the fullest extent possible.

10.6 Biohazard Containment

As exposure to infectious pathogens can occur through contact with contaminated needles, blood, blood products and respiratory specimens, appropriate precautions will be employed by all personnel during the collection, handling and processing of specimens, as recommended by the U.S. Centers for Disease Control and Prevention (CDC). Biohazardous waste will be contained according to institutional, transportation/carrier, and all other applicable regulations.

10.7 Storage of Specimens

Specimens collected during the course of this research will not be stored beyond a predefined maximum storage time of five years after collection, nor utilized for other purposes beyond

those specified in this protocol. Any leftover samples not consumed beyond this timeline will be destroyed.

11 POSSIBLE CONSTRAINTS

Anticipated implementation challenges to the successful outcome of the study include:

1. Ensuring quality and consistency of implementation of the study at two very different study sites. We plan to provide standardized training, supervision, and oversight to ensure quality and harmonized study procedures.

2. Enrolling the specified number of children with chest indrawing at each study site within the anticipated timeline. To address this concern, we will conduct the trial at high-volume health facilities to maximize enrollment. We will also aid recruitment by conducting community sensitization and outreach activities.

3. CXR and LUS interpretation bias. To eliminate interpretation bias, we will train panels of CXR and LUS experts to interpret all images. The panels will be blinded to each other's interpretations, and will adjudicate discordant interpretations in a process modeled after the WHO CXR process.[67] LUS and CXR interpretation will be based on *a priori* guidance set forth in the LUS SOP and CXR SOP.

4. Ensuring that CXR and LUS imaging does not delay appropriate care. Enrolled children may include children with comorbidities, including HIV infection or exposure, severe acute malnutrition, malaria, or severe anemia. The observational study design takes this into account by ensuring that all enrolled children will receive standard care. Extreme care will be taken to ensure that no necessary treatment is delayed to accommodate the imaging requirements.

5. Following up all children. Recognizing that some caregivers may not come back with their children for the follow-up visits, we plan to only enroll children who live close to the study site or are part of an ongoing demographic surveillance platform, and thus are more easily traceable or able to access the study site for follow-up. We will also train study staff to locate children who miss their follow-up appointments. We will ensure that study staff take the time to educate caregivers on the importance of follow-up.

6. Harmonizing the data between study sites. Because the study sites and the populations the hospital facilities serve are so different from each other, and the sample sizes of the enrolled children will be relatively small at each study site, of particular concern is that it will be difficult to harmonize the data between sites. To mitigate this risk, we are applying

the same enrollment criteria between study sites and will be training PLUS staff at both sites according to the same standardized protocols. We will also monitor PLUS study staff performance with multiple quality control procedures and interim trainings.

12 STUDY CHRONOGRAM

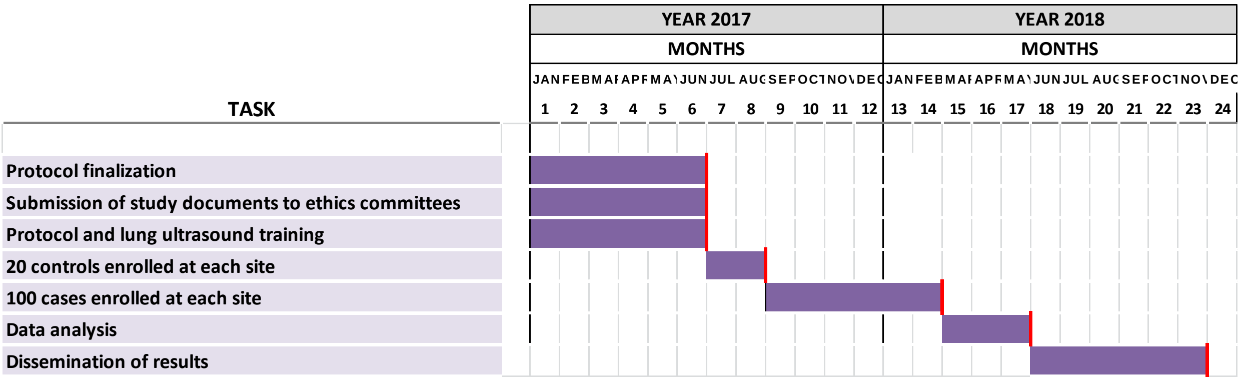

APPENDICES

Appendix I: Study Flow Diagram

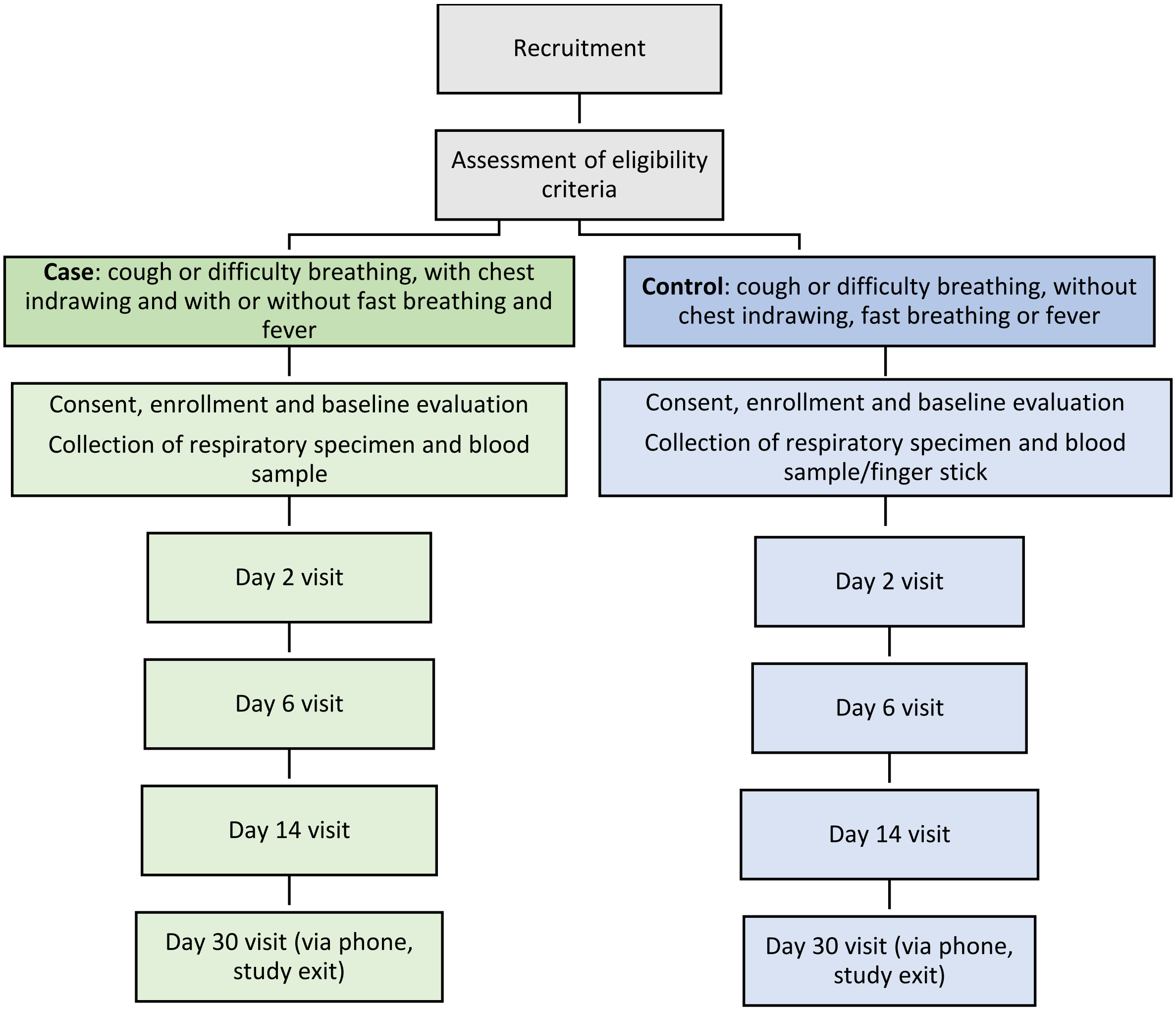

Appendix II: Schedule of Study Visits and Evaluations

Activity	Day 1: Screening and Enrollment	Day 2*	Day 6*	Day 14**	Day 30 (phone)**	Unscheduled
Eligibility Assessment	✓					
Informed Consent	✓					
Comprehension Checklist	✓					
Assign Participant ID	✓					
Demographics	✓					
Environmental Exposures	✓					
Locator Information	✓	✓	✓	✓		✓
Medical History	✓	✓	✓	✓	✓	✓
Vaccination History	✓					
Concomitant Medications/Antibiotic Use	✓	✓	✓	✓	✓	✓
Current Illness	✓	✓	✓	✓	✓	✓
Pneumonia Hospitalization	✓	✓	✓	✓		✓
Targeted Physical Exam	✓	✓	✓	✓		✓
Assess for Danger Signs	✓	✓	✓	✓		✓
Assess SAM	✓					
Perform CXR	✓					
Perform LUS	✓	✓	✓	✓		✓
Collect Respiratory Specimen***	✓					
Collect Blood Sample	✓					
Refer to Clinical Care (as needed)	✓	✓	✓	✓	✓	✓
Reimbursement	✓	✓	✓	✓		✓
Schedule Next Visit	✓	✓	✓			
End of Study Questions					✓	

* Window: +/- 24 hours
** Window: +/- 72 hours days
*** At investigator discretion

Appendix III: Specific Information Regarding the Manhiça site

Note: This Appendix is applicable to the **Mozambique site only**.

Rationale for this annex: The preceding protocol includes the generic information developed by the principal investigator for the PLUS study, regarding the overall activities to be conducted at each site being part of the project. However, understanding that each site may have its own particular idiosyncrasies, and as an attempt to clarify locally-specific details for Mozambique, we have drafted this specific annex summarizing in more detail procedures planned to occur at the Manhiça site as part of the PLUS project. This information cannot be described in more detail in the generic protocol as that document is meant to provide general explanations applicable to all PLUS sites.

A. The Manhiça site: A background summary

The study, coordinated by the Centro de Investigação em Saúde da Manhiça (CISM) in collaboration with the Barcelona Institute of Global health (ISGlobal) will be conducted in the District of Manhiça (population 163,000 inhabitants, 2300 km^2), a rural area located 90 km away from the capital Maputo. CISM was created in 1996 with the objective of conducting biomedical research in those diseases that affect the most poor and vulnerable. Manhiça is the paradigm of a poor, resource-constrained rural sub-Saharan African setting, with a population predominantly young (18% of which less than 5 years of age)[1]. CISM has been running a Demographic Surveillance System (DSS) since the year 1996, covering the whole district's population which includes a full census regularly updated of the population covered, and a detailed registry of all major demographic events (births, deaths, pregnancies, in- and out-migrations). For the purpose of this study, two demographic rounds will be conducted annually, covering the totality of the district's population. Additionally, CISM put in place in 1998 a morbidity surveillance system at Manhiça District Hospital (MDH) and 5 other peripheral health posts[2], to document pediatric morbidity and mortality. The enlarged DSS area includes one further hospital and 6 additional health posts (see Figure 1). Morbidity surveillance includes the systematic collection (using standardized forms) of demographic, clinical history, clinical exam, outcome and treatments for all children <15 years of age visiting the outpatient department or being admitted to hospital. Data on over 70,000 pediatric admissions and > 1 million outpatient visits have been collected over the past 18 years. Malaria screening (for all children with fever or a history of fever in the preceding 24 hours) and microbiological surveillance are also routinely conducted, and blood cultures are systematically collected for all admissions <2 years of age, and for older children with suspected severe disease. Over the past 15 years, CISM has conducted a series of studies with important impact on public health policies in the country, including studies on malaria preventive tools (RTS,S malaria candidate vaccine[3]; IPTi/IPTp[4, 5]), the treatment of malaria[6, 7], and the detailed description of the burden and epidemiology of childhood bacterial infections (including meningitis) in children with acute respiratory symptoms[8-12], as the basis for Mozambique's application for *Haemophilus Influenzae b* and Pneumococcal vaccines at GAVI. The Centre includes a fully equipped laboratory (including

parasitology, hematology, biochemistry, microbiology, (including biosafety level III premises), molecular biology (including PCR and RT-PCR) and immunology. The site has a dedicated freezer room, with twelve -80ºC freezers. Contamination rates in the past years have ranged between 5-13% of all processed blood cultures[13].

<u>Manhiça District Hospital (MDH)</u>

The Manhiça District Hospital (MDH), upgraded in 2011 from the Manhiça Health Centre, is the referral health facility for the entire Manhiça District. This public hospital includes a 32-bed pediatric ward, an 8-bed basic intensive care facility, and a day hospital (6-bed) where children can be temporarily admitted and observed prior to a final admission decision.

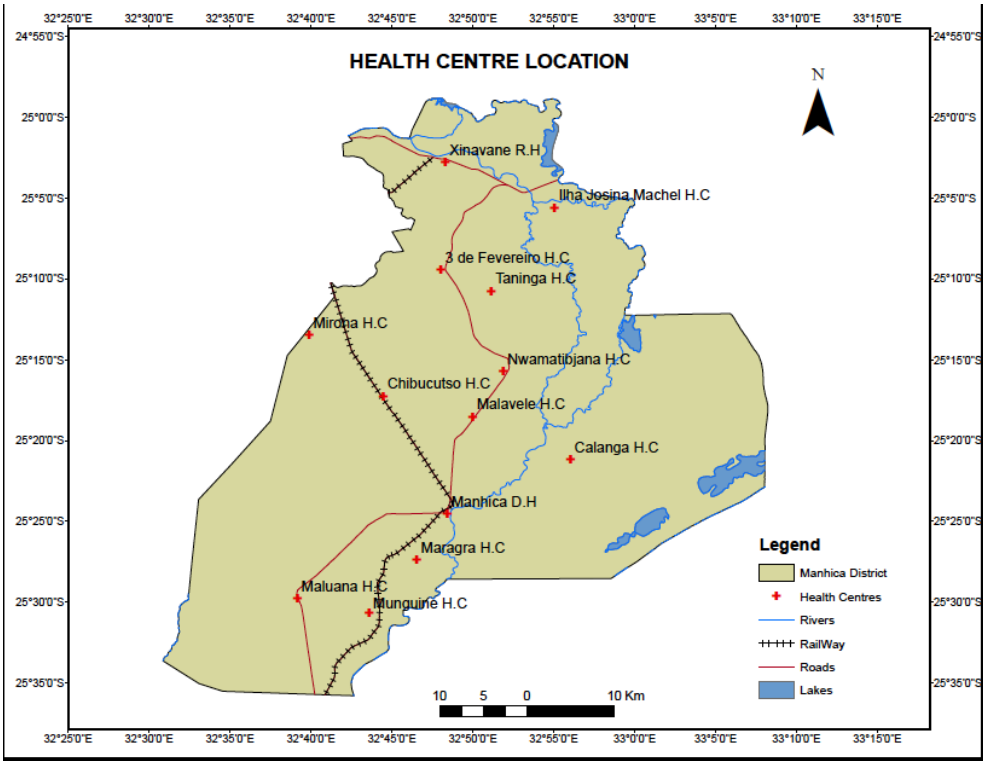

Figure 1: Map of the Manhiça district, and of the different health services available in the study area

The hospital has a maternity ward, a surgery room (where cesarean sections can be performed, together with basic emergency surgery), a fully digital (film-free) X-ray machine, and a clinical trials unit. It has been estimated that around 85% of the deliveries in the area (+/-5000 per year) are institutional deliveries, and a facility ("waiting home") is available at MDH for pregnant women with risk factors for a complicated delivery to settle by the hospital in attendance of labor, facilitating a supervised delivery. MDH's morgue, which has been recently refurbished, is where Minimally Invasive Tissue Sampling (MITS) procedures are routinely conducted for deaths

occurring at the Manhiça District, as part of the CHAMPS project general procedures. The morgue has two dissection tables, and 9 body fridges for the conservation of corpses.

Morbidity and mortality in the study area

By linking the information obtained through its morbidity surveillance system to the demographic data available for the DSS area, CISM has provided detailed descriptions of the health status of the community.

Latest estimates of HIV prevalence in community surveys of adults in Manhiça (39.9% seropositivity[14]) confirm the remarkably high burden imposed by this infection, and in recent years, a cohort of around 4,000 HIV-positive children has been routinely followed at the HIV outpatient consultation in MDH. CISM has also conducted etiological surveillance for the most common infections affecting children and infants in the area. Bacteremia rates peaked at $1730/10^5$ child-years at risk in infants less than one year old, $782/10^5$ in those 1–4 years old, and $49/10^5$ in children aged 5 years and older. The three main causes of invasive bacterial disease in newborns (<28 days of life) included: *Staph. aureus* (39% of cases), *Group B streptococcus* (GBS, 20%), *E. coli* (6%). *Haemophilus influenzae type B* and *S. pneumoniae* were also found to be important causes of bacteraemia in children 30-60 days[13]. Additionally, the main causes of invasive bacterial disease in non-neonate infants included *S. pneumoniae* (23%), *Non-typhoidal salmonella* (23%), *E. coli* (13%), *Hib* (13%) and *S. aureus* (8%)[13]

Pneumonia in the study area

Pneumonia surveillance has routinely been conducted in Manhiça since the year 2003, documenting the epidemiology, etiology, burden of disease, and clinical and radiographical characteristics of pneumonia in the area[9, 15-22]. Additionally, evaluations of the clinical overlap between malaria and pneumonia (of bacterial or viral origin)[12] have also been conducted, trying to ascertain relevant characteristics that may allow a more robust diagnostic differentiation of these entities, including host biomarker discovery[23, 24]. As surveillance started before the implementation of the two highly effective conjugate vaccines (i.e Hib vaccine, introduced in 2010; and antipneumococcal vaccine introduced in 2013), and continues as of today, it has been also possible to measure the public health impact of the introduction of these tools. Currently, the pneumonia surveillance ongoing at MDH includes the routine use of blood culture for all admitted children under the age of 2 years, and for those older than 2 with clinical signs of severity. Besides this, patients under the age of 5 years admitted to hospital with WHO-defined clinical signs of severe pneumonia, are routinely screened for nasopharyngeal carriage of Streptococcus pneumoniae, and HIV; and a Chest X ray is performed. All collected samples (blood, and NP samples) are routinely processed at CISM's laboratory for identification of bacteria using standard laboratory procedures. Antimicrobial susceptibility testing are performed for all bacteria isolates including pneumococci for patient management. Pneumococcal serotyping and other molecular of pneumococci are also performed in Manhiça laboratory. Pneumonia surveillance at MDH is part of a Project with the title "***Assessing***

Pneumococcal Conjugate Vaccine Impact On Pneumococcal Colonization, Disease And Deaths In Children <5 Years Of Age In Maputo, Mozambique", whose PI is Dr Betuel Sigaúque, co-investigator in the PLUS protocol.

For the purpose of te PLUS study, X-rays performed to children recruited will be interpreted centrally according to specific study procedures, similarly to those X-rays obtained from children recruited at the Pakistani site.

B. Rationale for each sample collected:

One of the objectives of this study is "to investigate whether there are different characteristic LUS imaging patterns between viral, bacterial and mixed pneumonia." In order to understand whether LUS produces imaging patterns that differ between each of these underlying etiologies, it is critical to adequately characterize what is the most likely underlying etiology for each enrolled child's pneumonia. The inclusion criteria for this study (cough and/or difficulty breathing with chest indrawing) have low specificity and can occur as a consequence of viral or bacterial infections. For this reason, one of the activities of this study investigates and seeks to characterize the underlying etiology of the pneumonia. Each sample has been selected to help determine the underlying etiology:

- Nasopharyngeal aspirate: Mucus obtained through this sampling method will be analyzed using a multiplex PCR molecular assay (FilmArray panel). This panel will screen for the presence of up to 20 different pathogens, namely:

Viruses
Adenovirus
Coronavirus HKU1
Coronavirus NL63
Coronavirus 229E
Coronavirus OC43
Human Metapneumovirus
Human Rhinovirus/ Enterovirus
Influenza A
Influenza A/H1
Influenza A/H1-2009
Influenza A/H3
Influenza B
Parainfluenza 1
Parainfluenza 2
Parainfluenza 3
Parainfluenza 4
Respiratory Syncytial Virus

Bacteria
Bordetella pertussis
Chlamydophila pneumoniae
Mycoplasma pneumoniae

- Nasopharyngeal swab: This sample, collected as part of the pneumonia surveillance ongoing in Manhiça, aims to evaluate carriage of pneumonia-associated bacteria, such as *Streptococcus pneumoniae*.

- Blood: A maximum of 3.5mL of blood will be collected upon admission. The sample will be divided into 1-2mL for blood culture (currently conducted as part of the invasive bacterial disease surveillance) and the remaining 1.25mL for full blood cell count (250 microlitres) and biomarker determination (1mL). In patients with bacterial pneumonia, blood culture is considered the gold standard methodology (even if "imperfect" in children) to confirm the diagnosis of invasive bacterial disease (IBD). The site in Manhiça has a recognized track record of bacterial disease surveillance for IBD. The remaining blood sample will be utilized for malaria diagnosis (blood slides), a full blood cell count, and biomarker evaluation. A predefined set of serum biomarkers with known or suspected etiologic and prognostic association will be assessed at a centralised laboratory in Barcelona for further characterization of the etiology and likely prognosis of each individual case. Biomarkers to be explored will include the following: C-reactive protein (CRP); Chitinase 3-like-1 (CHI3L1), soluble Tie2 receptor (sTie-2); endoglin, P-selectin; Procalcitonin (PCT), Angiopoeitin I and II (AngI; AngII); sTREM-1; Lipocalin (NGAL); and Von Willebrand factor (vWF). Finally, 2 drops of blood will be collected into a filter paper for molecular testing for malaria and/or bacterial infection.

C. Management, processing and storage of study samples obtained in Manhiça

Human samples derived from the performance of this study will be obtained from participating pediatric patients seen at Manhiça District Hospital and/or any of its linked health posts, within the Manhiça District.

<u>Sample management at CISM:</u>

All blood samples taken from patients within the study will be obtained through fingerprick or venous puncture, and always after a full explanation of the study has been provided and an informed consent form signed by the parents or guardians.

At the time of enrolment, and only if the child's clinical condition allows it (otherwise the child would not be included so as not to interfere with clinical care), <u>the following samples may be obtained</u>:

- 1 to 2 ml of vein blood for bacterial culture.
- 1.25 ml of vein blood in EDTA microtainers to perform a full blood cell count and for biomarker analysis. 250 ul will be used for blood cell count. The remaining plasma will be used for biomarker analysis after blood centrifugation and stored at -80ºC until

shipment to the centralized laboratories (Barcelona, Spain).

- 2 drops of blood into a whatmann filter paper, for microbiology (malaria and bacterial screening) molecular analyses
- 2 blood slides (for thin and thick film preparations) for malaria diagnosis
- Nasopharyngeal aspirate to perform viral and bacterial determination of respiratory pathogens
- Nasopharyngeal (NP) swab to evaluate carriage of bacterial pathogens

**Sample collected**	**Type of bodily fluid studied**	**Purpose of sample**	**Moment of sampling**	**Volume**	**Max volume**	**Part of ongoing pneumonia surveillance at MDH?**
Nasopharyngeal aspirate	Mucus	Resp. Viral/bacterial screening	Recruitment (admission)	2-3mL mucus		No
Nasopharyngeal swab	Nasopharyngeal exudate	Bacterial carriage	Recruitment (admission)	1 Swab		**Yes**
Blood	Full Blood	Culture of blood	Recruitment (admission)	1-2mL	3.5mL blood	**Yes**
	Plasma	Host response biomarkers	Recruitment (admission)	1 mL		No
	Full blood	Malaria slides	Recruitment (admission)	2 drops		No
	Full blood	Malaria/bacteria PCR	Recruitment (admission)	2 drops		No
	Full blood	White blood cell count	Recruitment (admission)	250microlitre		No

<u>The following laboratory analyses may be performed locally at CISM's laboratories</u>

- Hematocrit will be measured using a microcentrifuge and a Hawksley hematocrit reader card (Hawksley & Sons Ltd, Lancing, UK).
- Thick and thin blood films for malaria screening will be prepared, air-dried, Giemsa-stained, and examined using a light microscope fitted with a 10x oil immersion lens. Slides will be declared negative only after 200 fields have been read. Parasite numbers will be converted to a count/µl by assuming a standard leukocyte count of 8000/µl.
- Blood cultures will be performed by inoculating 1 to 3 mL of whole blood in a pediatric blood culture bottle and incubating it in an automated system for four days.
- NP swab will be cultured for screening of bacterial pathogen carriage.
- Serotyping of pneumococcal isolates (from any source) detected through the ongoing pneumonia surveillance

- Antimicrobial sensitivity of pathogens detected through the IBD surveillance or of pneumococcal isolates detected through the NP swab
- Full blood count will be performed in the CISM hematology laboratory.

The following laboratory analyses may be performed centrally at the centralized laboratories (Barcelona, Spain)

All the laboratory analyses related to screening of chemokine, cytokine and deeper characterization of the infectious agent, and finally the screening for presence of candidate biomarkers in patient blood, will be performed centrally at the Barcelona Institute for Global health (ISGlobal). Samples will be sent in batches according to a pre-specified shipment plan to the following address:

ISGlobal (Institut de Salut Global de Barcelona)
C/ Rosselló, 149 -153
Edifici CEK - 1ª planta
08036 Barcelona
Spain
Phone: +3493 227 54 00 (ext: 3388)
Contact person: Laura Puyol

Study samples from the Manhiça site sent to ISGlobal will be stored for a maximum of five years from the moment of reception. The study is expected to last for twelve months once the first child is recruited, however, the study may be extended and delays in processing some samples may occur. After five years, all samples not analysed will be destroyed. Remaining samples stored in Manhiça will be also maintained on site for a maximum of five years.

C. HIV-related procedures in Mozambican site

In Manhiça, Mozambique, HIV pre-test counseling, testing and post-test counseling will be done following WHO/UNAIDS guidelines and recommendations:

Pre-test counseling of the parent of a child admitted to hospital will be done by a VCT-trained HIV nurse/counselor. Major points of pre-test counseling will include:

- Explanation of why HIV testing is being recommended for these children and adults
- Voluntary nature of testing
- Testing procedures
- Interpretation of positive tests and requirement for further confirmatory test (PCR) if children are <18 months
- Implication of results (HIV-infected child most likely means mother is infected)
- Confidentiality of results
- Care being offered if their children test positive (clinic visits, CTX prophylaxis, HAART)
- Coping with results

- Availability of counseling, testing and care for the parent

After counseling, consent to do HIV testing on the child will be requested from the parents. As indicated in the informed consent, in the event of children being antibody positive, parents will be referred to VCT for their own counseling and for brothers/sisters of the study children.

HIV testing

HIV testing for study children with HIV status unknown will be performed after pre–test counseling of parents and only if consent has been obtained. Testing will be completed as follows:

- A finger prick for all study children with HIV status unknown will be performed to collect a small blood sample in a capillary for immediate HIV rapid testing.
- Children ≤ 18 months of age with positive rapid testing, two blood spots will be collected on filter paper for PCR testing.

Rapid testing will be done by a trained HIV counselor using *Determine®* as initial discriminatory diagnostic test, and *Unigold®* as a confirmatory test after a first positive result. These are the test kits and procedures approved by the Mozambique Ministry of Health according to the Guidelines for Using HIV Testing Technologies in Surveillance (EHO/CDS/CSR/EDC/2001.16).

For study purposes, the interpretation of HIV testing will be as follows:

In children < 18 months of age:

- If the *Determine®* test is negative, the child will be considered HIV negative. The *Unigold®* test will not be performed. No PCR will be necessary.
- If both rapid tests are positive, the child will be considered HIV antibody positive. Then confirmatory PCR test will be performed on a filter blood spot according to the procedures below.
- If the *Determine®* test is positive and the *Unigold®* test is negative, the result will be considered undetermined. PCR will be performed on a filter blood spot.

A child with a positive DNA-PCR test will be considered HIV-infected for the study purposes. If the PCR is negative, the child will be considered HIV-uninfected and return to *Center for crianças em risco* (CCR, "at-risk child's centre") for follow up.

Parents/caretakers will be given an appointment date to return four weeks later for the results at the CCR according to national guidelines. If the parent/caretaker does not return for the result within four weeks of appointment, a field worker will go to the house to re-invite the parent/caretaker to return to the MDH for the results.

In children ≥ 18 months of age

- If both rapid tests are positive, the child will be considered HIV-infected.

- If the *Determine*® test is negative, the child will be considered HIV-uninfected.
- If discordant results are found, a PCR will be done to decide the outcome for study purposes.

Parents of HIV-antibody positive children will be offered counseling and HIV testing for themselves and the brothers/sisters of the study children, as follows standard procedure at the Manhiça District Hospital, not for research purposes. Previous experiences noted that the best approach to increase adherence to treatment is to include the core family into the treatment schedule. If they accept, they will be screened following standard CISM procedures:

- Parents will be tested using the same rapid test kits as children.
- HIV infection will be considered only when both antibody tests are positive.

If discordant results occur, they will be asked to return one month later for follow-up testing. Testing of brothers/sisters of study children will follow standard CISM procedures, according to their age (as indicated for study children, including PCR tests according to age requirements).

Post-test counseling will be done following WHO/UNAIDS guidelines (4-7), after the rapid test results have been obtained. Parents will be informed of the results, information will be provided regarding the need for further testing and emotional support, and follow-up care will be discussed. Major points of post-test counseling will include:

- Interpretation of a positive test
- Implication of results (HIV-infected child most likely means mother is infected)
- Confidentiality of results
- Care being offered if child tests positive (clinical visits, CTX prophylaxis, HAART)
- Coping with results
- Availability of counseling, testing and care for the parent and brothers/sisters.

The Mozambican national pediatric criteria for initiating HAART are as follows:

- All children <5 years should start ART regardless of clinical stage and CD4 counts.
- Children > 5 years:
    - Symptomatic (clinical categories III or IV WHO)
    - Asymptomatic or symptomatic with CD4 counts <350.
    - Positive for tuberculosis (TB) or hepatitis B viral infection regardless of clinical stage and CD4 counts.

Mozambique's implementation of the new globally recommended ART guidelines may imply that children of any age may be started on ART immediately upon HIV diagnosis. Following national recommendations, CD4 counts will also be periodically performed in the CISM laboratory.

The current national recommendation for **first line pediatric HAART (<5 years old)** includes:

- Two nucleoside reverse transcriptase inhibitors (NRTI) (Lamivudine (3TC) and Zidovudine (ZT)) and one nonnucleoside reverse transcriptase inhibitor (Nevirapine (NVP)).
- First line pediatric HAART for children < 2 years old exposed to Nevirapine during pregnancy includes: 3TC, AZT and a protease inhibitor (Ritonavir/Lopinavir (LPVr)).
- According to national guidelines, second line pediatric HAART includes: Stavudine (D4T), 3TC and NVP or D4T, 3TC and LPVr (for children < 2 years old exposed to Nevirapine).

The current protocol will also take into account changes to national treatment guidelines as they are approved by the Mozambican government.

Children will be eligible for ART if they meet the requirements for compliance established in Mozambique. These criteria will be evaluated by a HAART committee at the Manhiça District Hospital and CISM. The requirements are as follows:

- The understanding that only HAART prescribed to the patient will be provided.
- A witness to supervise and follow up all the HAART treatment.
- Access to adequate supply of food and water in the house.
- Appropriate storage space for keeping medicines.
- Access to health post and follow up visits.
- Knowledge of proper means of self-administering the medicines.

Efforts will be made by counselors at CISM to assist the parents to meet these necessary criteria to provide treatment for their children.

Care for HIV-exposed, uninfected children

Children who initially have positive HIV antibody tests but have a PCR test will be considered as exposed and at risk of infection during the breastfeeding period. If the child becomes symptomatic at any time before reaching the age of 18 months, a new PCR test will be encouraged.

These children will be offered an outpatient visit at the special care clinic every two months, which will include:

- Clinical history and medical examination to identify possible symptoms of HIV infection which would have been acquired through breastfeeding.
- Mothers will voluntary receive nutritional education to minimize risk of infection to children through breastfeeding.
- If mothers choose not to breastfeed, artificial milk will be provided until the child reaches 12 months of age.

Following national guidelines, a repeat antibody test will be performed at 18 months to confirm or rule out infection if it has not yet been confirmed by PCR at that point. If the test at 18 months is negative, children will no longer need special care in the outpatient clinics. Parents will continue to be eligible to receive treatment and follow-up care at the HIV clinic.

Care for HIV-infected brothers/sisters of study children

Criteria for clinical follow-up and treatment for opportunistic infections and HAART (if indicated) are as outlined above for study children.

Care for HIV-infected parents

Parents will be followed every two months in the outpatient clinic at the Manhiça District Hospital by the same clinician and HIV nurse/counselor. According to national guidelines, the parents will be offered the same care and clinical follow-up as indicated below.

- An initial clinical history and medical examination for opportunistic infections and clinical follow-up for the subsequent visits.
- Cell blood count (CBC) checked prior starting CTX, one month after initiation and subsequently every six months. CTX prophylaxis will begin when the patient reaches a lymphocyte count <1,2X10^9/L and will continue throughout the lifetime. Screening for possible CTX toxicity will be performed and CTX will be stopped if appearance of drug-related rashes, allergies, hepatic or renal toxicity occurs, or if neutrophil count is < 0.5 X 10^9/L or anemia (< 8 g/dl).
- Mantoux test done to detect a possible TB latent infection. Any test >5mm of induration is considered to be positive, and in the absence of a positive radiological image or other suspicion of active TB, the patient will be started on isoniazid prophylaxis (300mg/daily) for six months. If active TB is suspected, diagnostic procedures will take place (baciloscopy, thorax X-ray) and, if necessary, TB treatment will be initiated, following the TB National Guidelines. Active case detection for TB is continuously performed.

Training for Personnel

The principal investigators will train study personnel in the method of patient enrollment and specimen collection. In collaboration with the Ministry of Health HIV/AIDS program, specific training on HIV counseling methods will be provided to clinicians and nurse/counselors in charge of counseling and care.

The project employs one counselor trained by MOH Mozambique and the CDC Global AIDS Program (GAP)/Mozambique in HIV counseling and testing. This counselor was recruited from VCT training programs in Maputo and is under the supervision of the coordinator of the HIV counseling and testing program. The supervisor will also ensure that results are kept confidential. HIV counselors are trained to perform the rapid HIV antibody tests.

HIV-PCR

HIV-PCR will be performed using the Roche HIV-1 DNA test, a qualitative in vitro test for the detection of HIV-1 in human blood. The test utilizes amplification of target DNA by PCR and nucleic acid hybridization for the detection of HIV-1 DNA. DNA will be extracted and amplified from dried blood spots on filter paper. Positive and negative control blood spots on filter paper will run in every PCR reaction.

Quality assurance will be periodically done using a panel of samples supplied by Roche and dried on filter paper to control for extraction procedures. Concurrent to the implementation of ARV treatment in Manhiça, an external quality control scheme will be set up for the PCR testing, according to national procedures.

HIV Data

Results of HIV tests will be entered into a restricted database containing study numbers only and no individual identifying information. Linkage between HIV test results and medical records containing identifiable information will only be done by study investigators in Manhiça and will remain in a locked file. No personally identifiable information will be collected for parents, brothers or sisters of study children.

Appendix III References

1. Sacoor C, Nhacolo A, Nhalungo D, et al. Profile: Manhica Health Research Centre (Manhica HDSS). International journal of epidemiology **2013**; 42(5): 1309-18.
2. Guinovart C, Bassat Q, Sigauque B, et al. Malaria in rural Mozambique. Part I: children attending the outpatient clinic. Malar J **2008**; 7: 36.
3. Alonso PL, Sacarlal J, Aponte JJ, et al. Efficacy of the RTS,S/AS02A vaccine against Plasmodium falciparum infection and disease in young African children: randomised controlled trial. Lancet **2004**; 364(9443): 1411-20.
4. Macete E, Aide P, Aponte JJ, et al. Intermittent preventive treatment for malaria control administered at the time of routine vaccinations in Mozambican infants: a randomized, placebo-controlled trial. The Journal of infectious diseases **2006**; 194(3): 276-85.
5. Menendez C, Bardaji A, Sigauque B, et al. A randomized placebo-controlled trial of intermittent preventive treatment in pregnant women in the context of insecticide treated nets delivered through the antenatal clinic. PloS one **2008**; 3(4): e1934.
6. Abdulla S, Sagara I, Borrmann S, et al. Efficacy and safety of artemether-lumefantrine dispersible tablets compared with crushed commercial tablets in African infants and children with uncomplicated malaria: a randomised, single-blind, multicentre trial. Lancet **2008**; 372(9652): 1819-27.
7. Bassat Q, Mulenga M, Tinto H, et al. Dihydroartemisinin-piperaquine and artemether-lumefantrine for treating uncomplicated malaria in African children: a randomised, non-inferiority trial. PloS one **2009**; 4(11): e7871.
8. Roca A, Bassat Q, Morais L, et al. Surveillance of acute bacterial meningitis among children admitted to a district hospital in rural Mozambique. Clinical infectious diseases : an official publication of the Infectious Diseases Society of America **2009**; 48 Suppl 2: S172-80.
9. Roca A, Sigauque B, Quinto L, et al. Estimating the vaccine-preventable burden of hospitalized pneumonia among young Mozambican children. Vaccine **2010**; 28(30): 4851-7.
10. Mandomando I, Sigauque B, Morais L, et al. Antimicrobial drug resistance trends of bacteremia isolates

in a rural hospital in southern Mozambique. The American journal of tropical medicine and hygiene **2010**; 83(1): 152-7.

11. Roca A, Quinto L, Abacassamo F, et al. Invasive Haemophilus influenzae disease in children less than 5 years of age in Manhica, a rural area of southern Mozambique. Tropical medicine & international health : TM & IH **2008**; 13(6): 818-26.
12. Bassat Q, Machevo S, O'Callaghan-Gordo C, et al. Distinguishing malaria from severe pneumonia among hospitalized children who fulfilled integrated management of childhood illness criteria for both diseases: a hospital-based study in Mozambique. The American journal of tropical medicine and hygiene **2011**; 85(4): 626-34.
13. Sigauque B, Roca A, Mandomando I, et al. Community-Acquired Bacteremia Among Children Admitted to a Rural Hospital in Mozambique. Pediatr Infect Dis J **2009**; 28(2): 108-13.
14. Gonzalez R, Munguambe K, Aponte J, et al. High HIV prevalence in a southern semi-rural area of Mozambique: a community-based survey. HIV medicine **2012**.
15. Annamalay AA, Lanaspa M, Khoo SK, et al. Rhinovirus species and clinical features in children hospitalised with pneumonia from Mozambique. Tropical medicine & international health : TM & IH **2016**; 21(9): 1171-80.
16. Bassat Q, Lanaspa M, Machevo S, et al. Hypoxaemia in Mozambican children <5 years of age admitted to hospital with clinical severe pneumonia: clinical features and performance of predictor models. Tropical medicine & international health : TM & IH **2016**; 21(9): 1147-56.
17. Lanaspa M, Annamalay AA, LeSouef P, Bassat Q. Epidemiology, etiology, x-ray features, importance of co-infections and clinical features of viral pneumonia in developing countries. Expert review of anti-infective therapy **2014**; 12(1): 31-47.
18. Lanaspa M, O'Callaghan-Gordo C, Machevo S, et al. High prevalence of Pneumocystis jirovecii pneumonia among Mozambican children <5 years of age admitted to hospital with clinical severe pneumonia. Clinical microbiology and infection : the official publication of the European Society of Clinical Microbiology and Infectious Diseases **2015**; 21(11): 1018.e9-.e15.
19. Lanaspa M, Valim C, Acacio S, et al. High reliability in respiratory rate assessment in children with respiratory symptomatology in a rural area in Mozambique. Journal of tropical pediatrics **2014**; 60(2): 93-8.
20. O'Callaghan-Gordo C, Bassat Q, Morais L, et al. Etiology and epidemiology of viral pneumonia among hospitalized children in rural Mozambique: a malaria endemic area with high prevalence of human immunodeficiency virus. Pediatr Infect Dis J **2011**; 30(1): 39-44.
21. Roca A, Sigauque B, Quinto L, et al. Invasive pneumococcal disease in children<5 years of age in rural Mozambique. Tropical medicine & international health : TM & IH **2006**; 11(9): 1422-31.
22. Sigauque B, Roca A, Bassat Q, et al. Severe pneumonia in Mozambican young children: clinical and radiological characteristics and risk factors. Journal of tropical pediatrics **2009**; 55(6): 379-87.
23. Diez-Padrisa N, Bassat Q, Machevo S, et al. Procalcitonin and C-reactive protein for invasive bacterial pneumonia diagnosis among children in Mozambique, a malaria-endemic area. PloS one **2010**; 5(10): e13226.
24. Valim C, Ahmad R, Lanaspa M, et al. Responses to Bacteria, Virus, and Malaria Distinguish the Etiology of Pediatric Clinical Pneumonia. American journal of respiratory and critical care medicine **2016**; 193(4): 448-59.

PROTOCOL REFERENCES

[1] Liu L, Oza S, Hogan D, Perin J, Rudan I, Lawn JE, Cousens S, Mathers C, Black RE. (2014) Global, Regional, and National Causes of Child Mortality in 2000-13, with Projections to Inform Post-2015 Priorities: An Updated Systematic Analysis. Lancet 385:430-440.

[2] World Health Organization. (2014) Integrated Management of Childhood Illness: Chart Booklet. Geneva, Switzerland: World Health Organization.

[3] United Nations Children's Fund Innovations. (2011) ARIDA (Acute Respiratory Infection Diagnostic Aid) [website]. New York, NY: United Nations Children's Fund. Date Accessed: May 13, 2015. https://innovateforchildren.org/projects/arida-acute-respiratory-infection-diagnostic-aid.

[4] Johansson E, Carvajal L, Newby H, Young M, Warlaw T. (2012) Pneumonia and Diarrhoea: Tackling the Deadliest Diseases for the World's Poorest Children. New York, NY.

[5] Rambaud-Althaus C, Althaus F, Genton B, D'Acremont V. (2015) Clinical Features for Diagnosis of Pneumonia in Children Younger Than 5 Years: A Systematic Review and Meta-Analysis. Lancet Infectious Disease 15:439-450.

[6] Zimmerman DR, Kovalski N, Fields S, Lumelsky D, Miron D. (2012) Diagnosis of Childhood Pneumonia: Clinical Assessment without Radiological Confirmation May Lead to Overtreatment. Pediatric Emergency Care 28:646-649.

[7] Andrews T, Thompson M, Buckley DI, Heneghan C, Deyo R, Redmond N, Lucas PJ, Blair PS, Hay AD. (2012) Interventions to Influence Consulting and Antibiotic Use for Acute Respiratory Tract Infections in Children: A Systematic Review and Meta-Analysis. PLOS One 7:e30334. PMCID: PMC3267713.

[8] Davies HD, Wang EE, Manson D, Babyn P, Shuckett B. (1996) Reliability of the Chest Radiograph in the Diagnosis of Lower Respiratory Infections in Young Children. Pediatric Infectious Disease Journal 15:600-604.

[9] World Health Organization. World Health Organization Essential Health Technologies: Strategy 2004-2007. Geneva, Switzerland.

[10] Hall EJ, Brenner DJ. (2008) Cancer Risks from Diagnostic Radiology. British Journal of Radiology 81:362-378.

[11] Moore CL, Copel JA. (2011) Point-of-Care Ultrasonography. New England Journal of Medicine 364:749-757.

[12] Levy JA, Noble VE. (2008) Bedside Ultrasound in Pediatric Emergency Medicine. Pediatrics 121:e1404-1412.

[13] Volpicelli G, Elbarbary M, Blaivas M, Lichtenstein DA, Mathis G, Kirkpatrick AW, Melniker L, Gargani L, Noble VE, Via G, Dean A, Tsung JW, Soldati G, Copetti R, Bouhemad B, Reissig A, Agricola E, Rouby JJ, Arbelot C, Liteplo A, Sargsyan A, Silva F, Hoppmann R, Breitkreutz R, Seibel A, Neri L, Storti E, Petrovic T. (2012) International Evidence-Based Recommendations for Point-of-Care Lung Ultrasound. Intensive Care Medicine 38:577-591.

[14] Darge K, Chen A. (2013) Ultrasonography of the Lungs and Pleurae for the Diagnosis of Pneumonia in Children: Prime Time for Routine Use. JAMA Pediatrics 167:187-188.

[15] Copetti R, Cattarossi L. (2008) Ultrasound Diagnosis of Pneumonia in Children. La Radiologia Medica 113:190-198.

[16] Shah VP, Tunik MG, Tsung JW. (2013) Prospective Evaluation of Point-of-Care Ultrasonography for the Diagnosis of Pneumonia in Children and Young Adults. JAMA Pediatrics 167:119-125.

[17] Ishrak TO. (2009) Ultrasound: A Revolution in the Making. Critical Ultrasound Journal 1:27-32.

[18] Lichtenstein DA, Lascols N, Meziere G, Gepner A. (2004) Ultrasound Diagnosis of Alveolar Consolidation in the Critically Ill. Intensive Care Medicine 30:276-281.

[19] Parlamento S, Copetti R, Di Bartolomeo S. (2009) Evaluation of Lung Ultrasound for the Diagnosis of Pneumonia in the Ed. American Journal of Emergency Medicine 27:379-384.

[20] Gehmacher O, Mathis G, Kopf A, Scheier M. (1995) Ultrasound Imaging of Pneumonia. Ultrasound in Medicine and Biology 21:1119-1122.

[21] Nazerian P, Volpicelli G, Vanni S, Gigli C, Betti L, Bartolucci M, Zanobetti M, Ermini FR, Iannello C, Grifoni S. (2015) Accuracy of lung ultrasound for the diagnosis of consolidations when compared to chest computed tomography. American Journal of Emergency Medicine 33:620-625.

[22] Cortellaro F, Colombo S, Coen D, Duca PG. (2012) Lung Ultrasound Is an Accurate Diagnostic Tool for the Diagnosis of Pneumonia in the Emergency Department. Emergency Medicine Journal 29:19-23.

[23] Caiulo VA, Gargani L, Caiulo S, Fisicaro A, Moramarco F, Latini G, Picano E. (2011) Lung Ultrasound in Bronchiolitis: Comparison with Chest X-Ray. European Journal of Pediatrics 170:1427-1433.

[24] Reissig A, Kroegel C. (2007) Sonographic Diagnosis and Follow-up of Pneumonia: A Prospective Study. Respiration 74:537-547.

[25] Reissig A, Copetti R, Mathis G, Mempel C, Schuler A, Zechner P, Aliberti S, Neumann R, Kroegel C, Hoyer H. (2012) Lung Ultrasound in the Diagnosis and Follow-up of Community-Acquired Pneumonia: A Prospective, Multicenter, Diagnostic Accuracy Study. Chest 142:965-972.

[26] Caiulo VA, Gargani L, Caiulo S, Fisicaro A, Moramarco F, Latini G, Picano E, Mele G. (2013) Lung Ultrasound Characteristics of Community-Acquired Pneumonia in Hospitalized Children. Pediatric Pulmonology 48:280-287.

[27] Weinberg B, Diakoumakis EE, Kass EG, Seife B, Zvi ZB. (1986) The Air Bronchogram: Sonographic Demonstration. American Journal Roentgenology 147:593-595.

[28] Lichtenstein DA, Meziere G, Seitz J. (2009) The Dynamic Air Bronchogram. A Lung Ultrasound Sign of Alveolar Consolidation Ruling out Atelectasis. Chest 135:1421-1425.

[29] Reissig A, Kroegel C. (2003) Transthoracic Ultrasound of Lung and Pleura in the Diagnosis of Pulmonary Embolism: A Novel Non-Invasive Bedside Approach. Respiration 70:441-452.

[30] Kim OH, Kim WS, Kim MJ, Jung JY, Suh JH. (2000) Us in the Diagnosis of Pediatric Chest Diseases. Radiographics 20:653-671.

[31] Chavez MA, Shams N, Ellington LE, Naithani N, Gilman RH, Steinhoff MC, Santosham M, Black RE, Price C, Gross M, Checkley W. (2014) Lung Ultrasound for the Diagnosis of Pneumonia in Adults: A Systematic Review and Meta-Analysis. Respiratory Research 15:50. PMCID: PMC4005846.

[32] Catalano D, Trovato G, Sperandeo M, Sacco MC. (2013) Lung Ultrasound in Pediatric Pneumonia. The Persistent Need of Chest X-Rays. Pediatric Pulmonology 49:617-618.

[33] Liu J, Liu F, Liu Y, Wang HW, Feng ZC. (2014) Lung Ultrasonography for the Diagnosis of Severe Neonatal Pneumonia. Chest 146:383-388.

[34] Ho MC, Ker CR, Hsu JH, Wu JR, Dai ZK, Chen IC. (2014) Usefulness of Lung Ultrasound in the Diagnosis of Community-Acquired Pneumonia in Children. Pediatric Neonatology 56:40-45.

[35] Hu QJ, Shen YC, Jia LQ, Guo SJ, Long HY, Pang CS, Yang T, Wen FQ. (2014) Diagnostic Performance of Lung Ultrasound in the Diagnosis of Pneumonia: A Bivariate Meta-Analysis. International Journal of Clinical and Experimental Medicine 7:115-121. PMCID: PMC3902248.

[36] Pereda MA, Chavez MA, Hooper-Miele CC, Gilman RH, Steinhoff MC, Ellington LE, Gross M, Price C, Tielsch JM, Checkley W. (2015) Lung Ultrasound for the Diagnosis of Pneumonia in Children: A Meta-Analysis. Pediatrics 135:714-722.

[37] Chavez MA, Naithani N, Gilman RH, Tielsch JM, Khatry S, Ellington LE, Miranda JJ, Gurung G, Rodriguez S, Checkley W. (2015) Agreement between the World Health Organization Algorithm and Lung Consolidation

Identified Using Point-of-Care Ultrasound for the Diagnosis of Childhood Pneumonia by General Practitioners. Lung [Epub Ahead of Print April 29, 2015].

[38] WINFOCUS. (2007) Article 1.3 Memorandum of Incorporation and Statute [Approved November 26, 2012]. Milan, Italy. http://www.winfocus.org/aboutus/vWoSon.

[39] Shah S, Noble VE, Umulisa I, Dushimiyimana JM, Bukhman G, Mukherjee J, Rich M, Epino H. (2008) Development of an Ultrasound Training Curriculum in a Limited Resource International Setting: Successes and Challenges of Ultrasound Training in Rural Rwanda. International Journal of Clinical and Experimental Medicine 1:193-196. PMCID: PMC2657276.

[40] Bedetti G, Gargani L, Corbisiero A, Frassi F, Poggianti E, Mottola G. (2006) Evaluation of Ultrasound Lung Comets by Hand-Held Echocardiography. Cardiovascular Ultrasound 4:34. PMCID: PMC1569440.

[41] Gargani L, Volpicelli G. (2014) How I Do It: Lung Ultrasound. Cardiovascular Ultrasound 12:25. PMCID: PMC4098927.

[42] Mercado-Young R, Martin DS, Caine T. (2010) Virtual Guidance: A New Technique to Empower Point-of-Care Ultrasound in Remote or Extreme Environments. Critical Ultrasound Journal 2:19-24.

[43] Spencer JK, Adler RS. (2008) Utility of Portable Ultrasound in a Community in Ghana. Journal of Ultrasound Medicine 27:1735-1743.

[44] Ma OJ, Norvell JG, Subramanian S. (2007) Ultrasound Applications in Mass Casualties and Extreme Environments. Critical Care Medicine 35:S275-279.

[45] McBeth PB, Hamilton T, Kirkpatrick AW. (2010) Cost-Effective Remote Iphone-Teathered Telementored Trauma Telesonography. Journal of Trauma 69:1597-1599.

[46] Zanobetti M, Poggioni C, Pini R. (2011) Can Chest Ultrasonography Replace Standard Chest Radiography for Evaluation of Acute Dyspnea in the Ed? Chest 139:1140-1147.

[47] Ali QM, Roth J, Abdel-Rahim IM, Franke D, Kardorff R, Schweisfurth H, Doehring E. (1999) Cost Saving with Ultrasonography in a Developing Country District Hospital. East African Medical Journal 76:272-274.

[48] Ross AB, DeStigter KK, Rielly M, Souza S, Morey GE, Nelson M, Silfen EZ, Garra B, Matovu A, Kawooya MG. (2013) A Low-Cost Ultrasound Program Leads to Increased Antenatal Clinic Visits and Attended Deliveries at a Health Care Clinic in Rural Uganda. PLOS One 8:e78450. PMCID: PMC3813603.

[49] Russell TC, Crawford PF. (2013) Ultrasound in the Austere Environment: A Review of the History, Indications, and Specifications. Military Medicine 178:21-28.

[50] Tsung JW, Kessler DO, Shah VP. (2012) Prospective Application of Clinician-Performed Lung Ultrasonography During the 2009 H1N1 Influenza a Pandemic: Distinguishing Viral from Bacterial Pneumonia. Critical Ultrasound Journal 4:16. PMCID: PMC3439382.

[51] Nascimento-Carvalho CM, Araujo-Neto CA, Ruuskanen O. (2015) Association between Bacterial Infection and Radiologically Confirmed Pneumonia among Children. Pediatric Infectious Disease Journal 34:490-493.

[52] United Nations Human Development Programme. (2015). Human Development Index (HDI) 205 Report. Retrieved from http://hdr.undp.org/en/content/human-development-index-hdi

[53] The World Bank. (2016). Mozambique. Retrieved from http://data.worldbank.org/country/mozambique

[54] The World Health Organization. (2016). Neonatal and Child Country Profile: Mozambique. Retrieved from http://www.who.int/maternal_child_adolescent/epidemiology/profiles/neonatal_child/moz.pdf

[55] Gonzalez R, Munguambe K, Aponte J, et al. (2012). High HIV prevalence in a southern semi-rural area of Mozambique: a community-based survey. HIV medicine.

[56] UNAIDS. (2016). HIV/AIDS estimates, Mozambique. Retrieved from http://www.unaids.org/en/regionscountries/countries/mozambique

[57] USAID. (2011). Demographic and Health Surveys (DHS), Mozambique. Retrieved from http://www.dhsprogram.com/what-we-do/survey/survey-display-362.cfm

[58] The World Bank (2016). Pakistan. Retrieved from http://data.worldbank.org/country/pakistan

[59] The World Health Organization (2016). Neonatal and Child Country Profile: Pakistan. Retrieved from http://www.who.int/maternal_child_adolescent/epidemiology/profiles/neonatal_child/pak.pdf?ua=1

[60] UNAIDS. (2016). HIV/AIDS estimates, Pakistan. Retrieved from http://www.unaids.org/en/regionscountries/countries/pakistan

[61] The Centers for Disease Control and Prevention. (2016). List of Malaria Endemic Countries. Retrieved from http://www.cdc.gov/travel-static/yellowbook/2014/map_3-10.pdf.

[62] The World Health Organization. (2015). Malaria Country Profile: Pakistan. Retrieved from http://www.who.int/malaria/publications/country-profiles/profile_pak_en.pdf?ua=1

[63] Connelly LM. (2008). Pilot studies. Medsurg Nursing, 17(6), 411-2.

[64] Hertzog MA. (2008). Considerations in determining sample size for pilot studies. Research in Nursing & Health, 31,180-191.

[65] Billingham SA, Whitehead AL & Julious SA. (2013). An audit of sample sizes for pilot and feasibility trials being undertaken in the United Kingdom registered in the United Kingdom Clinical Research Network database. BMC medical research methodology, 13(1), 1.

[66] Thabane L, Ma J, Chu R, et al. (2010). A tutorial on pilot studies: the what, why and how. BMC medical research methodology, 10(1), 1.

[67]Cherian T, Mulholland EK, Carlin JB, Ostensen H, Amin R, de Campo M, et al. Standardized interpretation of paediatric chest radiographs for the diagnosis of pneumonia in epidemiological studies. Bull World Health Organ. 2005;83(5):353-9.

[68]World Medical Association. (2002). World Medical Association Declaration of Helsinki: ethical principles for medical research involving human subjects. Journal of postgraduate medicine, 48(3), 206.
