# Supplementary material for: Multicentre pilot study evaluation of lung ultrasound for the management of paediatric pneumonia in low-resource settings: a study protocol
Source: BMJ Open Respir Res. 2018 Dec 19;5(1):e000340. doi: 10.1136/bmjresp-2018-000340 (PMC6307622; doi:10.1136/bmjresp-2018-000340)
Supplement: Supplementary data [file bmjresp-2018-000340supp002.pdf]

PLUS Lung Ultrasound Interpretation Form

**Enrollment ID:**		**Site:**	
**Interpreter Name:**		**Date of Interpretation:**	
**Visit:**	**[ ]Enrollment [ ]Day 2 [ ]Day 6 [ ]Day 14**		

Table 1. Interpretation of chest areas (check if YES, leave blank if NO)

**CHEST AREA**	Normal[1]	Interstitial Pattern[2]	Consolidation[3a]		Air bronchogram[4a]			Effusion			Unable to interpret due to insufficient length[6]	Unable to interpret due to poor quality scan	Not done or unavailable
			Small enough to measure: write diameter (cm) or UNK[3b]	Lobar[3c]	Dynamic[4b]	Static[4c]	Unknown if dynamic or static	Simple[5a]	Complex[5b]	Unknown if simple or complex			
RIGHT Anterior superior RA													
RIGHT Anterior inferior RA													
RIGHT Lateral superior RL													
RIGHT Lateral inferior RL													
LEFT Anterior superior LA													
LEFT Anterior inferior LA													
LEFT Lateral superior LL													
LEFT Lateral inferior LL													
RIGHT Posterior superior RP													
RIGHT Posterior inferior RP													
LEFT Posterior superior LP													
LEFT Posterior inferior LP													

Note: Each row should have at least one checked box.

Table 1 Definitions

1. **Normal**: pattern showing normal respiratory sliding (with or without A-lines) and without interstitial pattern, consolidation, air bronchogram or effusion
2. **Interstitial pattern**: pattern with three or more B-lines visible in the same moment in a single scan

3a. **Consolidation**: pattern with subpleural hypoechoic or tissue-like region with blurred margins and irregular shapes

3b. **Consolidation, small enough to measure**: if the consolidation is small enough that both edges are within the scan, measure the consolidation at the pleural base (Figure 1) and write the diameter (cm). If the consolidation is small enough to have both edges within the scan but you are unable to measure it for any reason, write 'UNK' (unknown).

3c. **Consolidation, lobar**: a consolidation that is too large to be measured in a single scan

4a. **Air bronchogram**: pattern in which the bronchi are visible either as a branching tree or small lentil-sized spots

4b. **Air bronchogram, dynamic**: an air bronchogram that moves relative to the consolidated lung synchronous with respiration

4c. **Air bronchogram, static**: all other air bronchograms should be considered static if not moving relative to the consolidated lung

5a. **Effusion, simple**: an anechoic (black) space between the two pleural layers

5b. **Effusion, complex**: a space between the two pleural layers showing any echoic (white) images inside (e.g., septa, debris)

6. **Unable to interpret due to insufficient length**: The clip is of insufficient length for interpretation. For clips using the probe in the longitudinal position, at least four intercostal spaces should be visible.

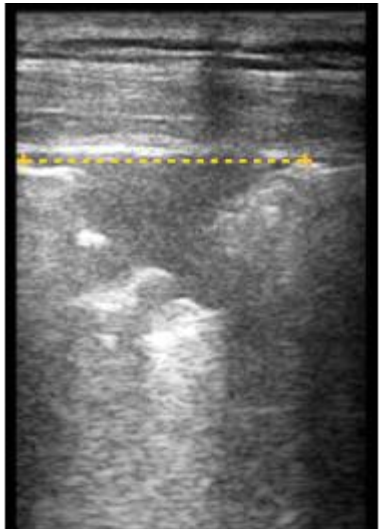

Figure 1. Measuring size of consolidation at the pleural base

---

Table 2. Overall conclusions (check if YES, leave blank if NO)

Overall conclusion	Right lung	Left lung
Normal		
Consolidation, lobar[1]		
Consolidation, peripheral[2]		
Obstructive atelectasis[3]		
Interstitial, focal[4]		
Interstitial, multifocal[5]		
Effusion, simple[6]		
Effusion, complex[7]		

Table 2 Definitions

1. **Consolidation, lobar**: a consolidation that is too large to be measured in a single scan
2. **Consolidation, peripheral**: a consolidation that is small enough to be measured and has air bronchogram(s) (dynamic or static) visible
3. **Obstructive atelectasis**: a consolidation that can be measured that does not have any air bronchogram(s)
4. **Interstitial, focal**: only one area (box) positive for an interstitial pattern
5. **Interstitial, multifocal**: Two or more areas (boxes) positive for interstitial patterns
6. **Effusion, simple**:an anechoic (black) space between the two pleural layers
7. **Effusion, complex**: a space between the two pleural layers showing any echoic (white) images inside (e.g., septa, debris)
